# Small-molecule cocktail 2SM promotes heart regeneration via inducing embryonic-like cardiomyocyte state

**DOI:** 10.1186/s13619-026-00301-0

**Published:** 2026-08-11

**Authors:** Zihao Wang, Lixia Zheng, Xiaokai Ma, Peng Gao, Yuanyuan Chen, Chenglu Xiao, Xin Zhao, Zhenyan Xiao, Xiaojun Zhu, Zhe Zhang, Jing-Wei Xiong

**Affiliations:** 1 Beijing Key Laboratory of Cardiometabolic Molecular Medicine, Institute of Molecular Medicine, and College of Future Technology, Peking University, Beijing, 100871 China; 2 School of Basic Medical Sciences, The Second Affiliated Hospital, Institute of Biomedical Innovation, The MOE Basic Research and Innovation Center for the Targeted Therapeutics of Solid Tumors, Jiangxi Medical College, Nanchang University, Nanchang, 330031 China; 3 Division of Cardiac Surgery, The Third Hospital of Peking University, Beijing, 100083 China

**Keywords:** Heart regeneration, Cardiomyocyte proliferation, Small molecule

## Abstract

Adult mammalian cardiomyocytes exhibit limited regenerative capacity, which remains a major challenge for functional recovery after myocardial infarction (MI). Our previous study identified a cocktail of five small molecules (5SM) that promotes cardiomyocyte proliferation and heart regeneration; however, the complexity of this five-compound mixture poses significant barriers to clinical translation and mechanistic studies. Here, we identified a simplified dual-compound cocktail (2SM) composed of phenylephrine hydrochloride (PE) and harmine (HM), which also robustly enhanced cardiomyocyte proliferation in vitro and in vivo. Transcriptomic and metabolomic analyses indicated that 2SM treatment activated cardiomyocyte cell-cycle programs and metabolic reprogramming. PE activated a fetal program and contributed to the up-regulation of CCND1 and WEE1 by activating mTOR signaling pathway, thus enabling cardiomyocytes to enter the G1/S phase but not G2/M phase. HM inhibited DYRK1A and activated MMB and FOXM1-MuvB complex, resulting in the upregulation of G2/M phase gene expression, of which the upregulated CDC25 proteins dephosphorylate CDK1, thereby relieving the inhibitory effect of PE. Sequential treatment of PE followed by HM was essential for completion of cytokinesis. In summary, we identified that 2SM, two compounds of 5SM, reprogrammed cardiomyocytes into an embryonic-like state, and precisely coordinated cell cycle progression for promoting cardiomyocyte proliferation.

**Supplementary Information:**

The online version contains supplementary material available at 10.1186/s13619-026-00301-0.

## Background

Ischemic heart disease (IHD) is the leading cause of human death worldwide (Naghavi et al. 2024). Following myocardial infarction caused by coronary artery occlusion, the heart loses a substantial number of cardiomyocytes due to ischemia and hypoxia (Del Buono et al. 2022). As adult cardiomyocytes possess extremely limited proliferative capacity, the damaged myocardium is gradually replaced by collagen-rich scar tissues, ultimately resulting in heart failure (Hashimoto et al. 2018). Although adult mammalian cardiomyocytes were long considered terminally differentiated and incapable of division, this dogma was re-shaped by the discovery that neonatal mammals can fully regenerate their hearts following injury (Porrello et al. 2011; Zhu et al. 2018). Lineage tracing studies have demonstrated that newly formed cardiomyocytes in neonatal hearts originate from pre-existing cardiomyocytes, indicating that mammalian cardiomyocytes retain intrinsic proliferative potential (Cui et al. 2020; Sereti et al. 2018). However, this regenerative ability of neonatal heart rapidly declines with postnatal maturation (Porrello et al. 2011; Zhu et al. 2018).

During early postnatal development, the cardiomyocyte microenvironment transitions from a hypoxic fetal state to a reoxygenated state, accompanied with a metabolic shift from glycolysis to mitochondrial oxidative phosphorylation (Puente et al. 2014; Sada and Kimura 2024). Notably, interventions that reduce oxygen levels, enhance glycolysis, or suppress mitochondrial metabolism have all been shown to improve cardiomyocyte proliferative capacity (Cardoso et al. 2020; Li et al. 2023; Nakada et al. 2017; Waypa et al. 2024). Besides, reprogramming adult cardiomyocytes into a fetal-like state using the OSKM transcription factor cocktail (*Oct4*, *Sox2*, *Klf4*, and *c-Myc*) has also been shown to promote cardiac regeneration (Chen et al. 2021). These observations suggest that reactivating an embryonic-like cardiomyocyte program is associated with heart regeneration. In addition to metabolic cues, cardiomyocyte proliferation is also regulated by Hippo/YAP pathway (von Gise et al. 2012; Liu et al. 2021a; Morikawa et al. 2017), microRNAs (Ouyang and Wei 2021), metabolic genes (Magadum et al. 2020; Fajardo et al. 2021), metabolites (Miklas et al. 2022; Shi et al. 2024), and cell cycle regulators (e.g., four factors including CCND1, CDK4, CCNB1 and CDK1) (Abouleisa et al. 2024; Abouleisa et al. 2022; Mohamed et al. 2018), highlighting the complex and multilayered control of cardiomyocyte cell cycle re-entry.

While several strategies have been proposed to reactivate cardiomyocyte proliferation, pharmacological manipulation using small-molecule compounds has emerged as a promising strategy to induce cardiomyocyte proliferation by targeting key regulators of the cell cycle (Ahmed et al. 2024; Kastan et al. 2022; Magadum et al. 2017; Mills et al. 2019). As small molecules are widely used in clinical practice and can be finely tuned for dose, timing, specificity and delivery, they offer a practical path toward therapeutic translation (Kinch et al. 2024). Our previous work has identified a five small-molecule cocktail (5SM), comprising an α1-adrenergic receptor agonist (phenylephrine hydrochloride, PE), a JAK1 inhibitor (baricitinib, BA), a dual-specificity tyrosine phosphorylation-regulated kinase 1 (DYRK1) family kinase inhibitor (harmine, HM), a PTEN inhibitor (VO-OHpic trihydrate, VO), and an MCT1 inhibitor (AZD3965), robustly promotes cardiomyocyte proliferation and cardiac regeneration in both neonatal and adult rats partially through the lactate-AARS2-mTORC1 axis (Du et al. 2022). Despite its high efficacy, the complexity of 5SM presents significant obstacles for therapeutic translation. Moreover, the individual contribution of each compound within the combination to cardiomyocyte proliferation remains unclear. Therefore, identifying a simplified and mechanistically defined small-molecule combination is crucial for advancing cardiomyocyte regeneration toward clinical application.

In this study, we identified a simplified dual-compound combination (2SM), consisting of PE and HM, which retains the pro-proliferative effects on cardiomyocytes. Mechanistically, PE induces a fetal-like program and activates mechanistic target of rapamycin complex 1 (mTORC1) signaling, promoting cardiomyocyte entry into G1/S phase. HM inhibits DYRK1A and activates the MMB (B-MYB-MuvB) and FOXM1-MuvB complex, leading to upregulation of G2/M-phase regulators. Together, 2SM cooperatively coordinates distinct phases of the cell cycle, enabling efficient cardiomyocyte cytokinesis.

## Results

### 2SM induces cardiomyocyte proliferation in vitro

**Fig. 1:**
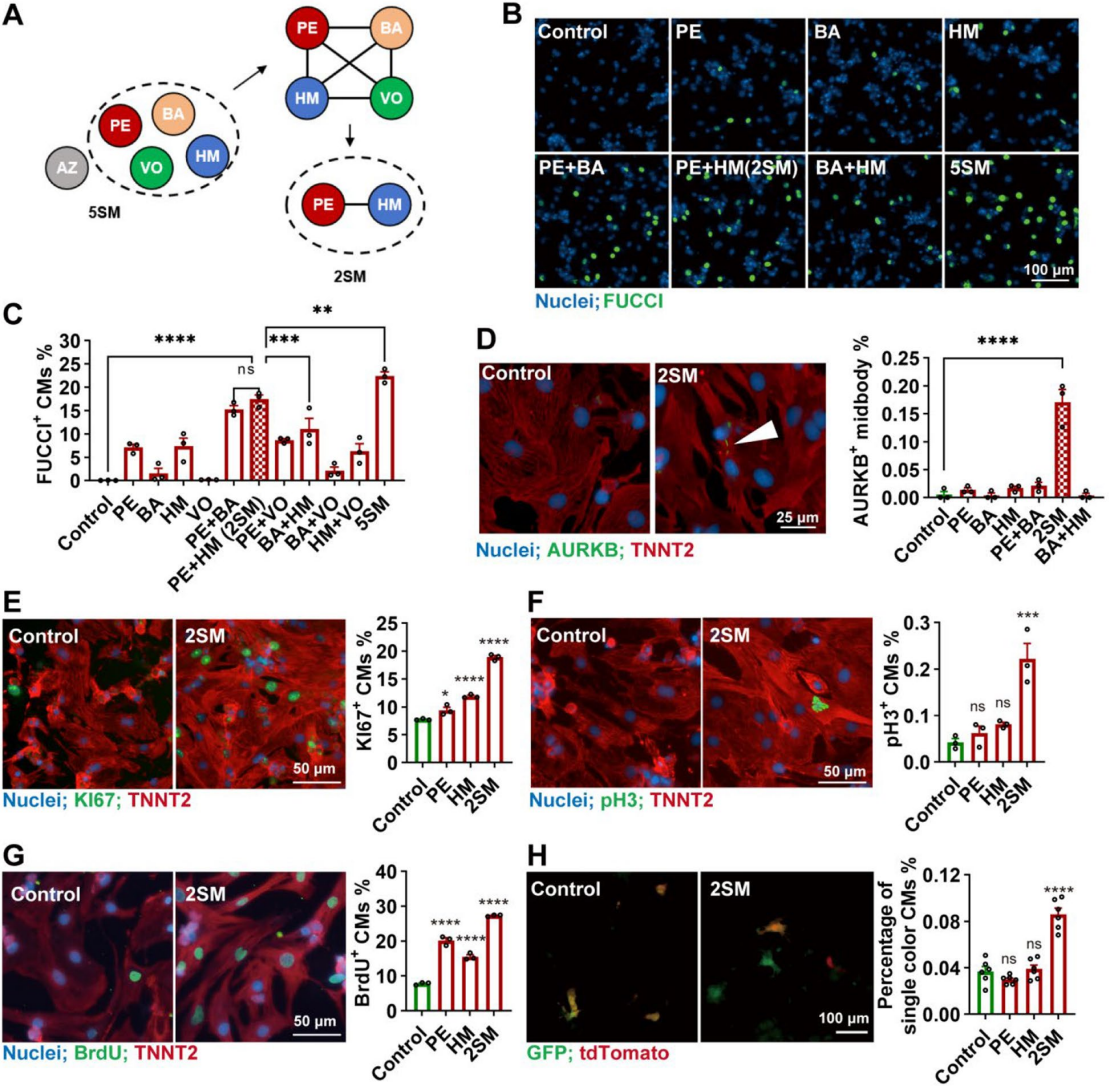
2SM promotes cardiomyocyte proliferation in vitro. **A** Schematic illustrating the screening strategy for simplified dual-compound combinations derived from the original five-compound cocktail (5SM). The 5SM formulation includes phenylephrine hydrochloride (PE), baricitinib (BA), harmine (HM), VO-OHpic trihydrate (VO), and AZD3965 (AZ). Excluding AZ, the remaining four compounds were tested in six pairwise combinations. Among them, the PE + HM combination (termed 2SM) was selected for its robust proliferative effect. **B** Representative images of FUCCI-positive P3 NRVMs. NRVMs were infected with Adeno-*Tnnt2*-FUCCI adenovirus and then treated with DMSO (control), single compounds or indicated compound combinations for 48 h. Scale bar, 100 μm. **C** Quantification of FUCCI-positive cardiomyocytes (CMs) from (**B**). *n* = 3 per group. Data represent mean ± SEM, with individual data points shown as dots overlaid on the bar graphs. Statistical analysis by one-way ANOVA (ns, not significant; **P < 0.01, ***P < 0.001, ****P < 0.0001). **D** Representative images and quantification of Aurora kinase B (AURKB)-positive midbodies in NRVMs after treatment with DMSO, single compounds or indicated compound combinations for 48 h. NRVMs were identified by TNNT2 (cardiac troponin T) staining (red). Scale bar, 25 μm. *n* = 3 per group. Data represent mean ± SEM, with individual data points shown as dots overlaid on the bar graphs. Statistical analysis by one-way ANOVA (****P < 0.0001). **E**–**G** Representative images and quantification of KI67-positive (**E**), pH3-positive (**F**) and BrdU-positive (**G**) NRVMs after treatment with DMSO, PE, HM or 2SM for 48 h. Scale bar, 50 μm. *n* = 3 per group. Data represent mean ± SEM, with individual data points shown as dots overlaid on the bar graphs. Statistical analysis by one-way ANOVA (ns, not significant; *P < 0.05, ***P < 0.001, ****P < 0.0001). **H** Representative images and quantification of single-colored MADM mouse cardiomyocytes. MADM cardiomyocytes were infected with Adeno-*Tnnt2*-Cre adenovirus and then treated with DMSO, PE, HM or 2SM for 72 h. Scale bar, 100 μm. *n* = 3 per group. Data represent mean ± SEM, with individual data points shown as dots overlaid on the bar graphs. Statistical analysis by one-way ANOVA (ns, not significant; ****P < 0.0001)

In our previous study on the 5SM cocktail, we found that four of the compounds, except AZD3965, were unable to induce cardiomyocyte cytokinesis when used individually (Du et al. 2022). Since AZD3965 alone had proliferative potential, it was studied separately, while the remaining four compounds were combined in pairs to generate six dual-compound combinations (Fig. 1A). To evaluate the efficiency of different dual-compound combinations in promoting cardiomyocyte proliferation, we employed the cardiomyocyte-specific FUCCI (fluorescent ubiquitination-based cell cycle indicator) reporter system, which labels cells in S, G2 and M phases (Sakaue-Sawano et al. 2008). Using the FUCCI system, we identified three combinations, PE + HM, PE + BA, and HM + BA, that most robustly increased the number of FUCCI-positive postnatal day 3 (P3) neonatal rat ventricular cardiomyocytes (NRVMs) compared to DMSO control (Fig. 1B-C). Since FUCCI labeling reflects cell cycle re-entry without confirming cytokinesis, we further immunostained Aurora kinase B (AURKB), a marker of late-stage mitosis. Among the three top combinations, only PE + HM (2SM) treatment showed the capacity to promote AURKB-positive midbody formation, suggesting that 2SM enhanced progression of cytokinesis (Fig. 1D).

In order to comprehensively validate the pro-proliferative effect, we next examined the 2SM-induced expression of other proliferation markers. Immunostaining revealed that 2SM treatment also increased the numbers of KI67-positive (Fig. 1E), phosphorylated histone H3 (pH3)-positive (Fig. 1F), and BrdU-incorporating (Fig. 1G) NRVMs, confirming the 2SM induction of cardiomyocyte cell cycle. Additionally, we utilized the mosaic analysis with double markers (MADM) mouse model to assess the actual cardiomyocyte division, where successful mitotic recombination results in single-colored cardiomyocytes following Cre-driven DNA recombination. We found that 2SM treatment significantly increased the number of single-colored cardiomyocytes compared to DMSO control, whereas neither individual PE nor HM treatment led to an increase in single-colored cardiomyocytes (Fig. 1H). Collectively, these results demonstrate that 2SM efficiently promotes cardiomyocyte proliferation and division in vitro.

To further address the translational relevance of 2SM in a human model system, we next examined whether 2SM could induce cell-cycle activation in human induced pluripotent stem cell-derived cardiomyocytes (hiPSC-CMs). Compared to DMSO control, 2SM treatment increased the percentage of both pH3-positive and KI67-positive hiPSC-CMs (Fig. S1), indicating 2SM promotes cell-cycle reactivation in an in vitro human cardiomyocyte model.

To evaluate whether 2SM treatment induces sustained cardiomyocyte proliferation, we assessed the proliferative effects of continuous versus transient 2SM treatment. P3 NRVMs were treated with 2SM for 48 h, after which the treatment was either continued or replaced with DMSO for an additional 48 h. Immunostaining showed that withdrawal of 2SM led to a marked reduction in KI67-positive, pH3-positive, and AURKB-positive cardiomyocytes, whereas continuous treatment maintained elevated levels of proliferating cardiomyocytes (Fig. S2). These findings indicate that 2SM stimulates transient cardiomyocyte cell cycle activity and does not induce permanent or autonomous cell proliferation.

### 2SM promotes cardiomyocyte proliferation and heart regeneration after myocardial infarction in vivo

**Fig. 2:**
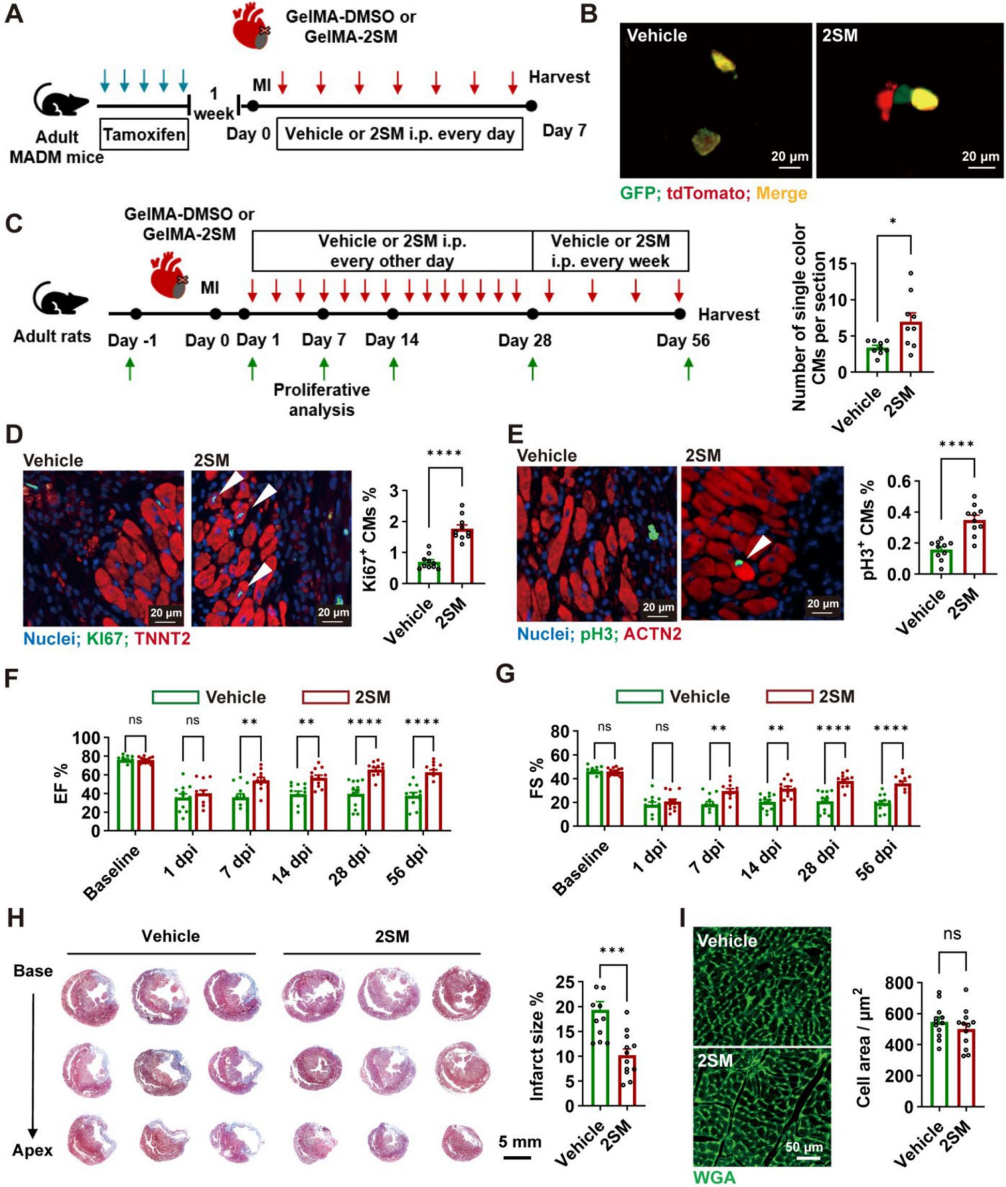
2SM promotes cardiomyocyte proliferation and heart regeneration after myocardial infarction (MI) in vivo. **A** Schematic of 2SM delivery and heart harvest in adult αMHC-MerCreMer; MADM mice after MI. Blue arrows represent the administration of Tamoxifen, red arrows represent the administration of vehicle or 2SM. **B** Representative images and quantification of single-colored cardiomyocytes in αMHC-MerCreMer; MADM mice at 7 day post injury (dpi) after administration of vehicle or 2SM. Scale bar, 20 μm. Vehicle: *n* = 9 animals; 2SM: *n* = 9 animals; analyzed from 3 sections per animal. Data represent mean ± SEM, with individual animals shown as dots overlaid on the bar graphs. Statistical analysis by Student’s* t*-test (*P < 0.05). **C** Schematic of 2SM delivery, echocardiography and heart harvest in adult rats after MI. Red arrows represent the administration of vehicle or 2SM, the time points at which echocardiography was performed to assess cardiac function**. D** and **E** Representative images and quantification of KI67-positive (D) and pH3-positive (**E**) cardiomyocytes in the border region at 7 dpi. Arrowheads indicate positive cardiomyocytes. Cardiomyocytes were identified by TNNT2 and ACTN2 (α-Actinin) staining (red). Scale bar, 20 μm. Vehicle: *n* = 10 animals; 2SM: *n* = 10 animals; analyzed from 3 sections per animal. Data represent mean ± SEM, with individual animals shown as dots overlaid on the bar graphs. Statistical analysis by Student’s *t*-test (****P < 0.0001). **F** and **G** Serial echocardiographic assessment of ejection fraction (EF%, F) and fractional shortening (FS%, G) from vehicle or 2SM-treated rats at multiple time points after MI. Vehicle: *n* = 12 animals; 2SM: *n* = 12 animals. Data represent mean ± SEM, with individual animals shown as dots overlaid on the bar graphs. Statistical analysis by Student’s *t*-test (ns, not significant; **P < 0.01, ****P < 0.0001). **H** Representative Masson's trichrome staining images from serial cross-sections (from base to apex) of rat hearts treated with vehicle or 2SM at 56 dpi, and quantification of infarct size. Scale bar, 5 mm. Vehicle: *n* = 12 animals; 2SM: *n* = 12 animals. Data represent mean ± SEM, with individual animals shown as dots overlaid on the bar graphs. Statistical analysis by Student's *t*-test (***P < 0.001). **I** Representative images and quantification of cardiomyocyte cross-sectional area measured by wheat germ agglutinin (WGA) staining at 56 dpi. Scale bar, 50 µm. Vehicle: *n* = 12 animals; 2SM: *n* = 12 animals. Data represent mean ± SEM, with individual animals shown as dots overlaid on the bar graphs. Statistical analysis by Student’s *t*-test (ns, not significant)

We next sought to determine whether 2SM similarly induced cardiomyocyte proliferation and heart regeneration in adult hearts following MI in vivo. To directly assess cardiomyocyte cytokinesis, we utilized αMHC-MerCreMer; MADM mice subjected to MI via permanent ligation of the Left anterior descending (LAD) coronary artery. Mice were pretreated with tamoxifen intraperitoneally for 5 consecutive days before MI. Immediately after MI, we applied GelMA-DMSO or GelMA-2SM hydrogel locally to the infarcted area of the left ventricle, followed by administration of either DMSO or 2SM for 7 consecutive days after MI (Fig. 2A). At 7 days post injury (dpi), 2SM treatment significantly increased the number of single-colored cardiomyocytes compared with vehicle, indicating enhanced cardiomyocyte cytokinesis (Fig. 2B).

To evaluate the long-term effects of 2SM on heart regeneration and function recovery, we established an MI model in adult rats. Animals were treated with GelMA-DMSO or GelMA-2SM immediately after MI, and received DMSO or 2SM every other day for the first 4 weeks, followed by weekly injections until 8 weeks post-MI (Fig. 2C). At 7 dpi, adult rats treated with 2SM showed a significant increase in the total number of isolated cardiomyocytes compared with vehicle controls (Fig. S3A-B). Analysis of nucleation revealed an increase in mononucleated CMs and a decrease in polyploid CMs (Fig. S3C-D). Immunostaining revealed that 2SM treatment increased percentage of KI67-positive (Fig. 2D) and pH3-positive cardiomyocytes (Fig. 2E) in the infarct border zone. Collectively, these data indicate 2SM induced cell cycle re-entry and increased cardiomyocyte number in adult MI hearts.

From 7 to 56 dpi, serial echocardiography demonstrated much improved cardiac function in 2SM-treated rats, with increased ejection fraction (EF) (Fig. 2F) and fractional shortening (FS) (Fig. 2G) compared to controls. Consistent with functional recovery, Masson’s trichrome staining showed a significant reduction in infarct size in 2SM-treated hearts compared to control hearts at 56 dpi (Fig. 2H). To further compare the efficacy of 2SM and 5SM, we assessed cardiac function in adult rats at 7 dpi. Both 2SM and 5SM treatments significantly improved EF and FS compared with vehicle controls, with no significant differences between the two treatment groups (Fig. S4), indicating 2SM and 5SM have comparable therapeutic efficacy in vivo.

Because pathological cardiac hypertrophy also leads to transient improvements in contractile function, we assessed cardiomyocyte size to distinguish heart regeneration from hypertrophic remodeling. We measured cardiomyocyte size in NRVMs following DMSO or 2SM treatment in vitro, and found no significant changes in cell area after 2SM treatment (Fig. S5A). Cardiomyocyte cross-sectional area measurements by wheat germ agglutinin (WGA) staining also showed no significant difference in cell area between DMSO and 2SM-treated groups at 7dpi and 56dpi in vivo (Fig. S5B, 2I). Collectively, these results demonstrate that 2SM promotes adult cardiomyocyte proliferation and improves heart function following MI, without triggering cardiomyocyte hypertrophy.

To exclude potential systemic toxicity of 2SM, we further assessed the morphology and organ-to-body weight ratios of the liver, spleen, lungs, and kidneys at 56 dpi. We found no significant differences between the vehicle and 2SM-treated groups in either organ morphology or organ-to-body weight ratio (Fig. S5C-D). Histological analysis by hematoxylin and eosin (H&E) staining revealed no detectable abnormalities in major organs at 56 dpi after 2SM treatment (Fig. S6A). In addition, serum alanine aminotransferase (ALT, Fig. S6B) and aspartate aminotransferase (AST, Fig. S6C) levels were comparable between Vehicle and 2SM groups at 56 dpi. Collectively, these results indicate that systemic 2SM administration does not induce detectable histological or biochemical toxicity in major organs.

### 2SM induces cardiomyocyte cell cycle activation and metabolic reprogramming

**Fig. 3:**
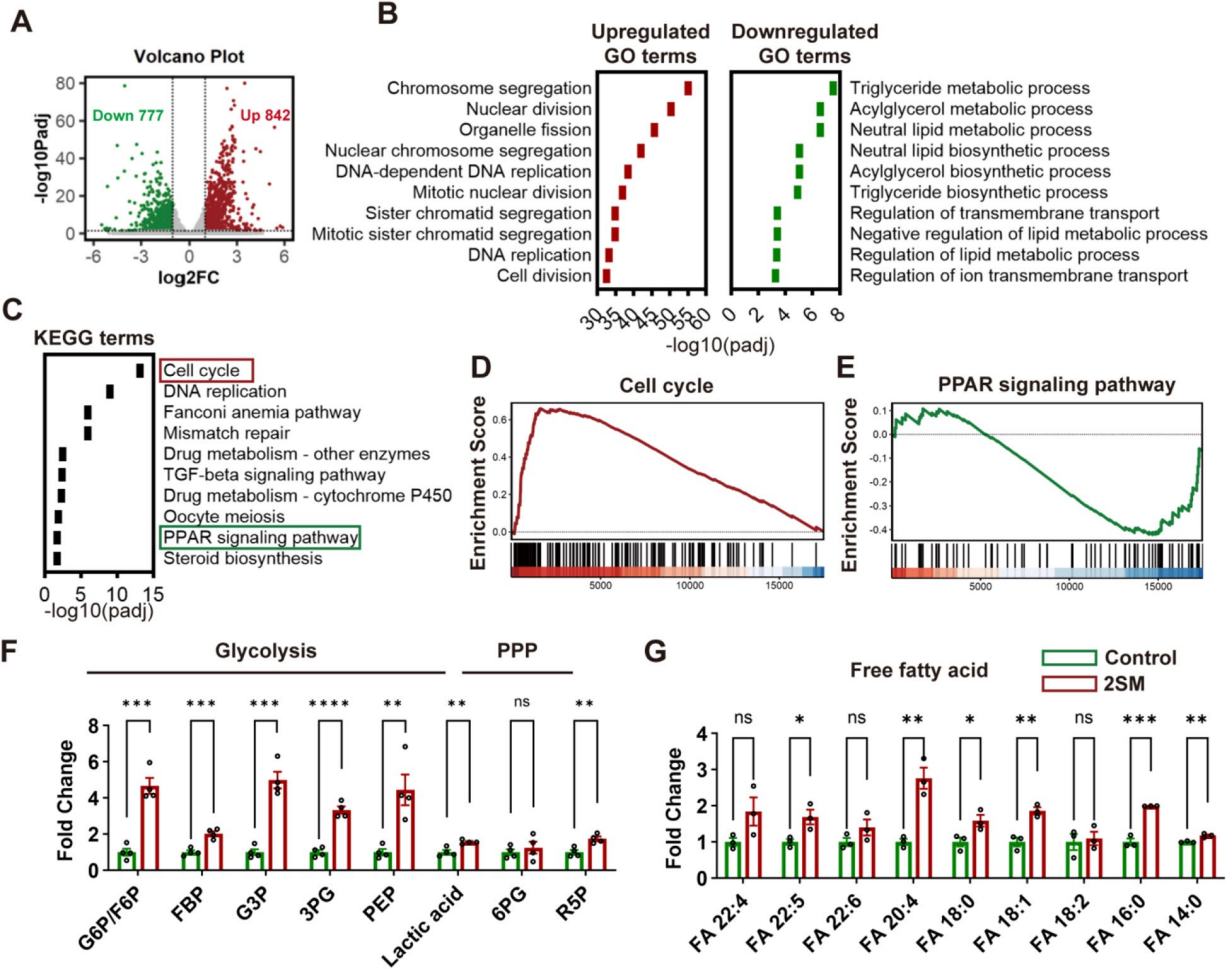
Transcriptome and metabolomic analyses reveal that 2SM activates cell cycle processes and metabolic switches. **A** Volcano plot of differentially expressed genes (DEGs) and unchanged genes in the 2SM-treated group versus the control group. Genes with adjusted *P* < 0.05 and |log_2_FC|> 1 are considered significant. *n* = 3 per group. **B** GO enrichment analysis of up-regulated (left) and down-regulated (right) genes in 2SM-treated group versus control group. **C** KEGG pathway enrichment analysis of DEGs in 2SM-treated group versus control group. **D** and **E** Gene set enrichment analysis (GSEA) plots show representative enriched KEGG pathways. **D** Cell cycle pathway is positively enriched in 2SM group. **E** PPAR signaling pathway is negatively enriched in 2SM group. **F** and **G** Metabolite profiling of glycolysis (**F**), pentose phosphate pathway (PPP, F), and free fatty acids (**G**) in NRVMs after treatment with 2SM or DMSO (Control) for 24 h. *n* = 4 per group. Data represent mean ± SEM, with individual data points shown as dots overlaid on the bar graphs. Statistical analysis by Student’s *t*-test (ns, not significant; *P < 0.05, **P < 0.01, ***P < 0.001, ****P < 0.0001)

To investigate the molecular mechanisms underlying 2SM-induced cardiomyocyte proliferation, we performed RNA sequencing (RNA-seq) analysis of P3 NRVMs treated with DMSO (control) or 2SM for 24 h. Differential expression analysis identified 842 upregulated and 777 downregulated genes in 2SM group compared to control group (Fig. 3A). Gene Ontology (GO) enrichment analysis of the upregulated genes showed a significant enrichment in biological processes related to cell cycle progression, including chromosome segregation, DNA replication and cell division (Fig. 3B, left panel). Besides, downregulated genes were enriched in metabolic processes, particularly those related to lipid and fatty acid metabolism (Fig. 3B, right panel). Consistently, Kyoto encyclopedia of genes and genomes (KEGG) pathway analysis confirmed activation of the cell-cycle and DNA replication pathways, and suppression of the peroxisome proliferator-activated receptor (PPAR) signaling pathway (Fig. 3C). Consistently, genes associated with the cell cycle, such as *Aurkb*, *Cdk1*, and *Cdc25c*, were markedly upregulated in 2SM-treated compared to control NRVMs (Fig. S7A), while key PPAR pathway regulators including *Ppara*, *Cpt1b*, and *Cpt1a*, were significantly downregulated (Fig. S7B). The PPAR signaling pathway plays a central role in regulating fatty acid uptake, β-oxidation, and lipid metabolism in cardiomyocytes (Montaigne et al. 2021). Downregulation of PPAR pathway components is consistent with the suppression of lipid and fatty acid metabolism upon 2SM treatment. Gene set enrichment analysis (GSEA) further supports these findings, showing positive enrichment of the cell cycle pathway (Fig. 3D) and a negative enrichment of the PPAR signaling pathway (Fig. 3E) in 2SM-treated NRVMs.

Previous studies have shown that proliferating cardiomyocytes exhibit increased glycolytic flux and decreased fatty acid oxidation, a metabolic phenotype that supports the biosynthetic and energetic demands of cell division (Sada and Kimura 2024). Based on our transcriptomic findings indicating downregulation of fatty acid metabolic pathways, we next performed metabolomic profiling to assess whether 2SM treatment induces similar metabolic shifts in proliferating cardiomyocytes. Metabolomic profiling revealed elevated levels of glycolytic and pentose phosphate pathway intermediates, including G6P/F6P, FBP, G3P, 3PG, PEP, lactic acid, and R5P in 2SM-treated NRVMs (Fig. 3F). In contrast, several long-chain fatty acids such as FA 20:4, FA 18:0, and FA 16:0 were increased in 2SM-treated cardiomyocytes compared to controls, indicating 2SM-induced inhibition of fatty acid catabolism (Fig. 3G). Free fatty acid profiling in myocardial tissue from vehicle- and 2SM-treated MI rats at 7 dpi also revealed a general trend toward increased levels of several fatty acids after 2SM treatment (Fig. S8).

Together, these results indicate that 2SM promotes cardiomyocyte proliferation by activating cell cycle programs while simultaneously inducing a metabolic shift characterized by enhanced glycolysis and suppressed fatty acid metabolism.

### PE induces fetal-like and hypertrophy-associated transcriptional priming, whereas HM promotes cardiomyocyte cell-cycle progression

Having established that 2SM induces pro-proliferative transcriptomic changes in cardiomyocytes, we next sought to deconvolute the individual contributions of PE and HM to this response. RNA-seq analysis showed that PE-treated cardiomyocytes displayed a transcriptional profile distinct from controls by principal component analysis (Fig. S9A). Differential expression analysis identified 383 upregulated and 283 downregulated genes following PE treatment (Fig. S9B). GO enrichment analysis of upregulated genes revealed enrichment of muscle hypertrophy-related terms, accompanied by increased expression of fetal/hypertrophy-associated genes, including *Nppa*, *Nppb*, and *Myh7* (Fig. S9C, left panel; Table S1). Consistently, GSEA showed positive enrichment of the physiological cardiac muscle hypertrophy gene set in PE-treated NRVMs compared with controls (Fig. S9D), supporting activation of hypertrophy-associated transcriptional signatures. RNA-seq-based quantification further demonstrated a significant increase in the *Myh7*/*Myh6* ratio after PE treatment (Fig. S9E), consistent with reactivation of a fetal-like transcriptional program. In parallel, downregulated genes were enriched in lipid and fatty acid metabolic processes, including *Cpt1a* and *Hmgcs2* (Fig. S9C, right panel; Table S2). Together, these data indicate that PE induces a fetal-like and hypertrophy-associated transcriptional priming state, accompanied by metabolic reprogramming.

In contrast, HM primarily activated a mitotic cell-cycle transcriptional program. HM-treated cardiomyocytes were clearly separated from controls by principal component analysis (Fig. S9F). Differential expression analysis identified 350 upregulated and 263 downregulated genes following HM treatment (Fig. S9G). GO enrichment analysis of upregulated genes revealed strong enrichment of cell-cycle-related biological processes, including chromosome segregation, nuclear division, sister chromatid segregation, and mitotic nuclear division (Fig. S9H; Table S3). Key G2/M regulators, including *Aurkb*, *Cdk1*, and *Prc1*, were markedly upregulated (Table S3), indicating that HM preferentially activates G2/M gene expression programs to promote mitotic progression.

We then asked whether the hypertrophy-associated transcriptional priming induced by PE is preserved as a full hypertrophic program in the combined 2SM context. Although fetal/hypertrophy-associated genes, including *Nppa*, *Nppb*, and *Myh7*, were upregulated in 2SM-treated NRVMs (Fig. S10A), and the *Myh7*/*Myh6* ratio was increased (Fig. S10B), neither GO enrichment analysis nor GSEA of the cardiac hypertrophic response gene set showed significant enrichment of hypertrophy-related programs in 2SM-treated NRVMs (Fig. 3B and Fig. S10C). Together with the unchanged CM size observed both in vitro and in vivo, these results suggest that 2SM redirects PE-induced fetal/hypertrophy-associated priming toward a proliferative transcriptional state, without inducing overt pathological hypertrophic growth.

### Sequential PE and HM treatment facilitates complete cardiomyocyte cell cycle progression

Transcriptomic profiling indicated that PE and HM play distinct roles in promoting cardiomyocyte proliferation. To further investigate how each compound regulates specific phases of the cardiomyocyte cell cycle, we first assessed key cell cycle regulators and checkpoint activation at the G1/S and G2/M phases.

**Fig. 4:**
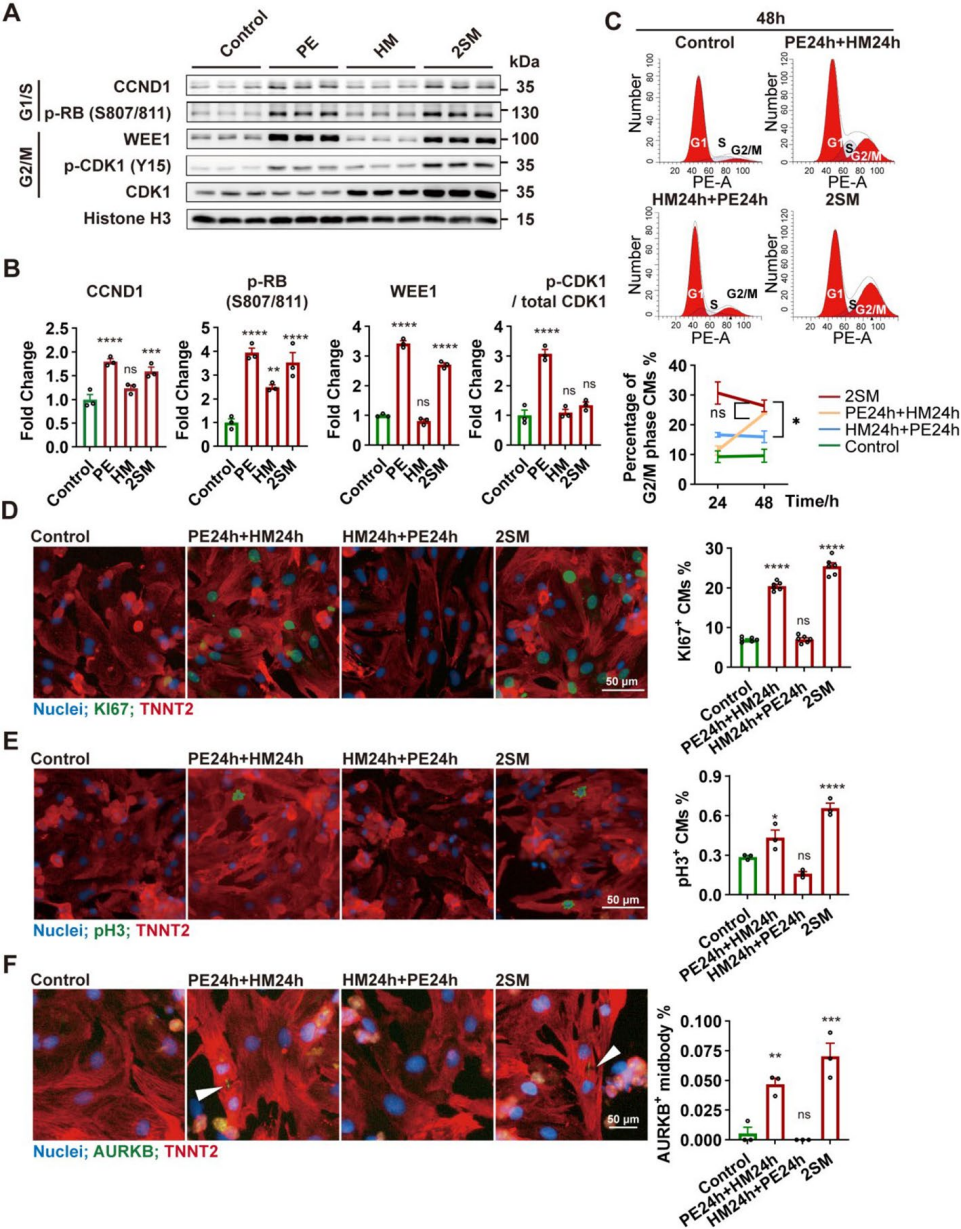
Sequential treatment of PE and then HM, but not vice versa, promotes cardiomyocyte cytokinesis. **A** Western blot analysis of key G1/S and G2/M phase regulators in NRVMs after treatment with DMSO (Control), PE, HM, or 2SM for 24 h. **B** Quantification of protein expression levels from (**A**). Data are normalized to Histone H3 and presented as fold changes relative to control. *n* = 3 per group. Data represent mean ± SEM, with individual data points shown as dots overlaid on the bar graphs. Statistical analysis by one-way ANOVA (ns, not significant; **P < 0.01, ***P < 0.001, ****P < 0.0001). **C** Flow cytometry analysis of cell cycle progression and quantification of G2/M-phase NRVMs following DMSO (Control), 2SM, and sequential PE + HM or HM + PE treatment. *n* = 3 per group. Data represent mean ± SEM. Statistical analysis by one-way ANOVA (ns, not significant; *P < 0.05). **D**-**F** Representative immunofluorescence images and quantification of KI67-positive (D), pH3-positive (**E**), and AURKB-positive (**F**) NRVMs following DMSO (Control), 2SM, and sequential PE + HM or HM + PE treatment. Scale bar, 50 μm. KI67: *n* = 6 per group. pH3 and AURKB: *n* = 3 per group. Data represent mean ± SEM, with individual data points shown as dots overlaid on the bar graphs. Statistical analysis by one-way ANOVA (ns, not significant; *P < 0.05, **P < 0.01, ***P < 0.001, ****P < 0.0001)

During the G1/S phase, cyclin D1 (CCND1) forms a complex with CDK4/6 to phosphorylate the retinoblastoma protein (RB) at multiple sites, including serine 807/811. Phosphorylation of RB leads to its functional inactivation, resulting in the release of E2F transcription factors. The liberated E2Fs then drive the transcription of genes essential for DNA replication and S phase entry, thereby promoting progression through the cell cycle (Fischer and Müller 2017). Immunoblotting revealed that PE treatment significantly upregulated CCND1 and p-RB levels, whereas HM alone had minimal effects. Notably, the 2SM treatment produced the strongest activation of these G1/S markers (Fig. 4A). We next assessed the G2/M checkpoint, where the kinase WEE1 inhibits cell cycle progression by phosphorylating CDK1 at tyrosine 15, thereby preventing premature mitotic entry. Immunoblotting showed that PE treatment increased WEE1 expression and p-CDK1 levels, thus repressing the activation of a G2/M checkpoint. In contrast, PE and HM co-treatment decreased p-CDK1 levels, indicating that HM relieved the G2/M arrest imposed by PE (Fig. 4B). These results suggest that PE and HM regulate distinct phases of the cardiomyocyte cell cycle, that is, PE primarily promotes cardiomyocyte entry into the cell cycle by facilitating the G1/S transition, but simultaneously represses G2/M checkpoint activation that prevents mitotic progression. In contrast, HM specifically acts to relieve the G2/M block and promote successful entry into mitosis.

To functionally validate these findings, we performed flow cytometry and immunostaining following sequential treatments with PE and HM. Cardiomyocytes treated with PE for 24 h followed by HM for another 24 h exhibited a significant increase in the proportion of cells in G2/M phase compared to the reverse sequence (Fig. 4C). Moreover, sequential PE and HM treatment significantly enhanced the expression of proliferation markers, including KI67 (Fig. 4D), pH3 (Fig. 4E), and AURKB-positive midbodies (Fig. 4F), reaching the pro-proliferative levels comparable to those induced by 2SM treatment. Collectively, these results indicate that PE initiates cardiomyocyte cell-cycle re-entry via G1/S activation but induces a G2/M checkpoint arrest. Subsequent HM treatment overcomes this checkpoint block, thereby enabling complete cell-cycle progression. Thus, sequential activation—first by PE, then by HM—is essential for efficient cardiomyocyte proliferation.

### mTORC1 activation is necessary but insufficient for PE-induced cardiomyocyte proliferation

**Fig. 5:**
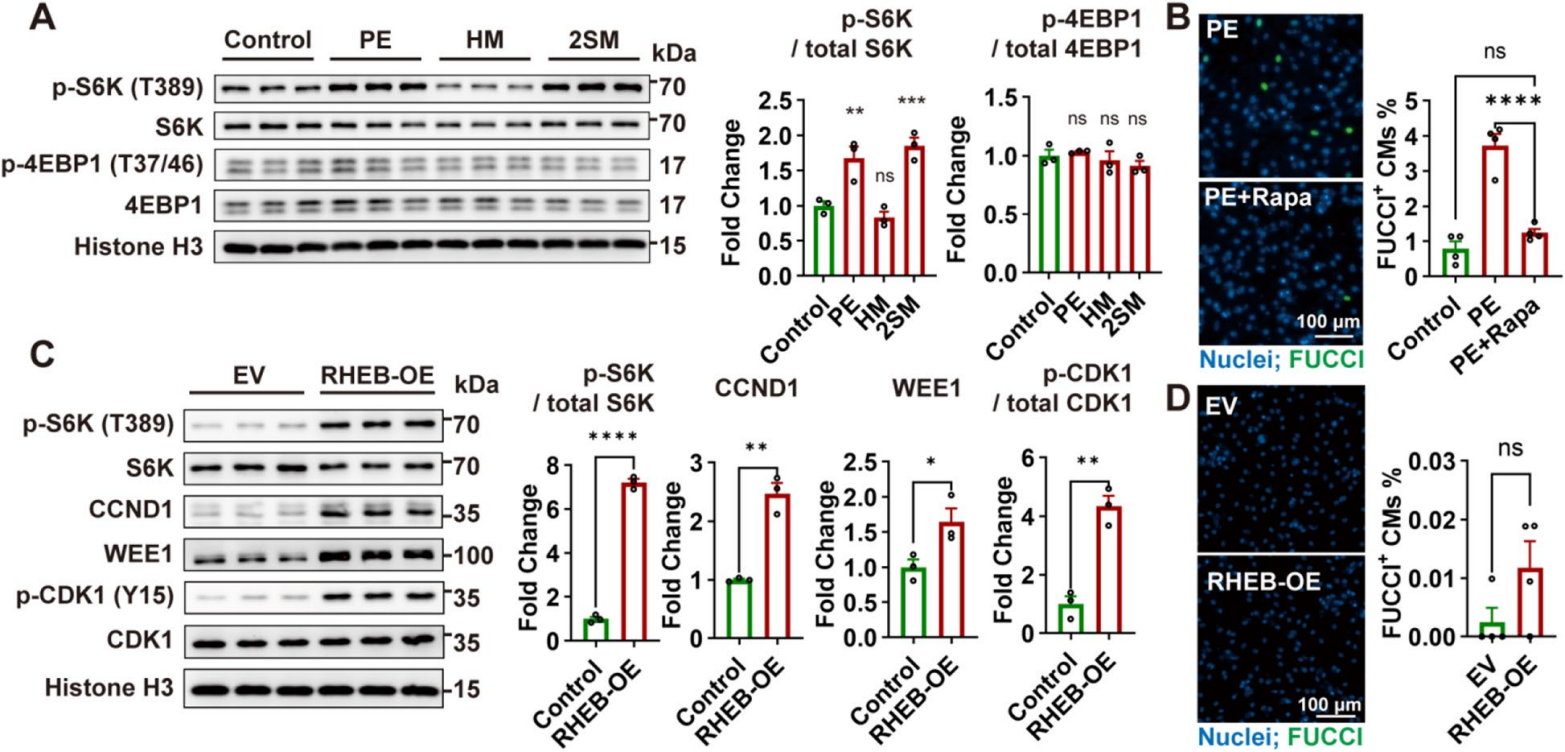
PE activates mTORC1-S6K signaling, which is necessary but insufficient for cardiomyocyte cell-cycle re-entry. **A** Western blot analysis of canonical mTORC1 downstream effectors in NRVMs treated with DMSO (control), PE, HM, or 2SM for 24 h. Bar graphs show quantification of p-S6K/S6K and p-4EBP1/4EBP1 ratios. Data are normalized to Histone H3 and presented as fold change relative to control. *n* = 3 per group. Data represent mean ± SEM, with individual data points shown as dots overlaid on the bar graphs. Statistical analysis by one-way ANOVA (ns, not significant; **P < 0.01, ***P < 0.001). **B** Representative immunofluorescence images and quantification of FUCCI-positive NRVMs after treatment with PE or PE plus rapamycin (Rapa). *n* = 3 per group. Scale bar, 100 μm. Data represent mean ± SEM, with individual data points shown as dots overlaid on the bar graphs. Statistical analysis by one-way ANOVA (ns, not significant; ****P < 0.0001). **C** Western blot analysis of NRVMs infected with Rheb-overexpressing adenovirus (RHEB-OE) or empty vector (EV) infection for 72 h, showing the protein levels of p-S6K/S6K, CCND1, WEE1, and p-CDK1/CDK1. Data are normalized to Histone H3 and presented as fold change relative to EV. *n* = 3 per group. Data represent mean ± SEM, with individual data points shown as dots overlaid on the bar graphs. Statistical analysis by Student’s *t*-test (ns, not significant; *P < 0.05, **P < 0.01, ****P < 0.0001). **D** Representative images and quantification of FUCCI-positive NRVMs after RHEB-OE or EV control infection for 72 h. *n* = 3 per group. Scale bar, 100 μm. Data represent mean ± SEM, with individual data points shown as dots overlaid on the bar graphs. Statistical analysis by Student’s *t*-test (ns, not significant)

Since PE and HM regulate distinct phases of the cardiomyocyte cell cycle, we next explored the upstream signaling pathways that might explain their differential effects on cell cycle regulation. Previous studies have revealed that mTORC1 signaling was activated in 5SM-treated cardiomyocytes (Du et al. 2022). To determine whether mTORC1 is also involved in the response to PE and HM treatment, we examined the phosphorylation status of canonical mTORC1 downstream effectors. Western blot analysis showed that PE or 2SM treatment, but not HM, significantly increased phosphorylation of S6 kinase (S6K), suggesting that PE is the primary contributor to mTORC1-S6K activation within the 2SM combination (Fig. 5A). Interestingly, the phosphorylation of 4EBP1, another downstream target of mTORC1, remained unchanged across all treatment groups. To functionally validate the role of mTORC1 in PE-mediated proliferation, we treated PE-stimulated cardiomyocytes with the mTORC1 inhibitor rapamycin. Inhibition of mTORC1 abolished the PE-induced increase in FUCCI-positive cardiomyocytes, confirming the necessity of mTORC1 activity for PE-mediated cell cycle re-entry (Fig. 5B).

To further determine whether activation of mTORC1 alone is sufficient to recapitulate the effects of PE, we overexpressed Rheb, a small GTPase that specifically activates mTORC1 signaling (Yang et al. 2017). As expected, Rheb overexpression (Rheb-OE) led to increased phosphorylation of S6K and upregulation of cell cycle regulators including CCND1, WEE1, and phosphorylated CDK1 (Fig. 5C), but failed to increase the proportion of FUCCI-positive cardiomyocytes (Fig. 5D), indicating that mTORC1 activation alone is insufficient to induce cardiomyocyte cell cycle re-entry. Previous transcriptomic profiling indicated that PE also induces a fetal-like gene expression program and suppresses fatty acid metabolism. We therefore compared the expression of representative fetal and metabolic genes following PE or Rheb-OE treatment. Quantitative PCR revealed that both PE and Rheb-OE significantly downregulated *Cpt1a* and *Cpt1b*, genes involved in fatty acid oxidation (Fig. S11A). However, only PE, but not Rheb-OE, induced the expression of the fetal cardiac genes *Nppa* and *Nppb* (Fig. S11B). Since Rheb activates mTORC1 but fails to induce fetal gene expression, this may explain why it is insufficient to trigger cell cycle re-entry on its own.

Together, these findings indicate that PE promotes cardiomyocyte cell cycle re-entry primarily through concurrent activation of mTORC1 and fetal gene expression. The activation of mTORC1 inhibits fatty acid metabolism and enhances protein translation, which leads to increased CCND1 protein levels. The elevated CCND1 facilitates G1/S transition and supports initial cell cycle activation. However, mTORC1 activation also induces WEE1 upregulation, which inhibits CDK1 activity and results in G2/M checkpoint arrest. These data suggest that while PE effectively primes cardiomyocytes for proliferation, additional signals such as those provided by HM are required to complete cell-cycle progression.

### HM activates MMB and FOXM1-MuvB complex to induce cardiomyocyte G2/M phase entry

Building on evidence that HM regulates G2/M phase genes, we next sought to investigate the upstream regulatory mechanism by which HM promotes cardiomyocyte G2/M phase progression. LIN52 is a core subunit of the MuvB complex, which associates with different partner proteins to form either the repressive DREAM complex or the transcriptionally active MMB and FOXM1-MuvB complexes. Previous studies have shown that phosphorylation of LIN52 by Dyrk1a is required for the assembly of the DREAM complex, which suppresses cell cycle gene expression (Iness et al. 2019; Litovchick et al. 2011). In contrast, accumulation of dephosphorylated LIN52 facilitates the formation of MMB and FOXM1-MuvB complexes (Iness et al. 2019; Litovchick et al. 2011), which are essential for the activation of G2/M-specific genes and mitotic progression. Based on these findings, we hypothesized that HM regulates cardiomyocyte G2/M phase by inhibiting DYRK1A-mediated LIN52 phosphorylation and enhancing MuvB-dependent activation of the G2/M gene transcriptional program.

**Fig. 6:**
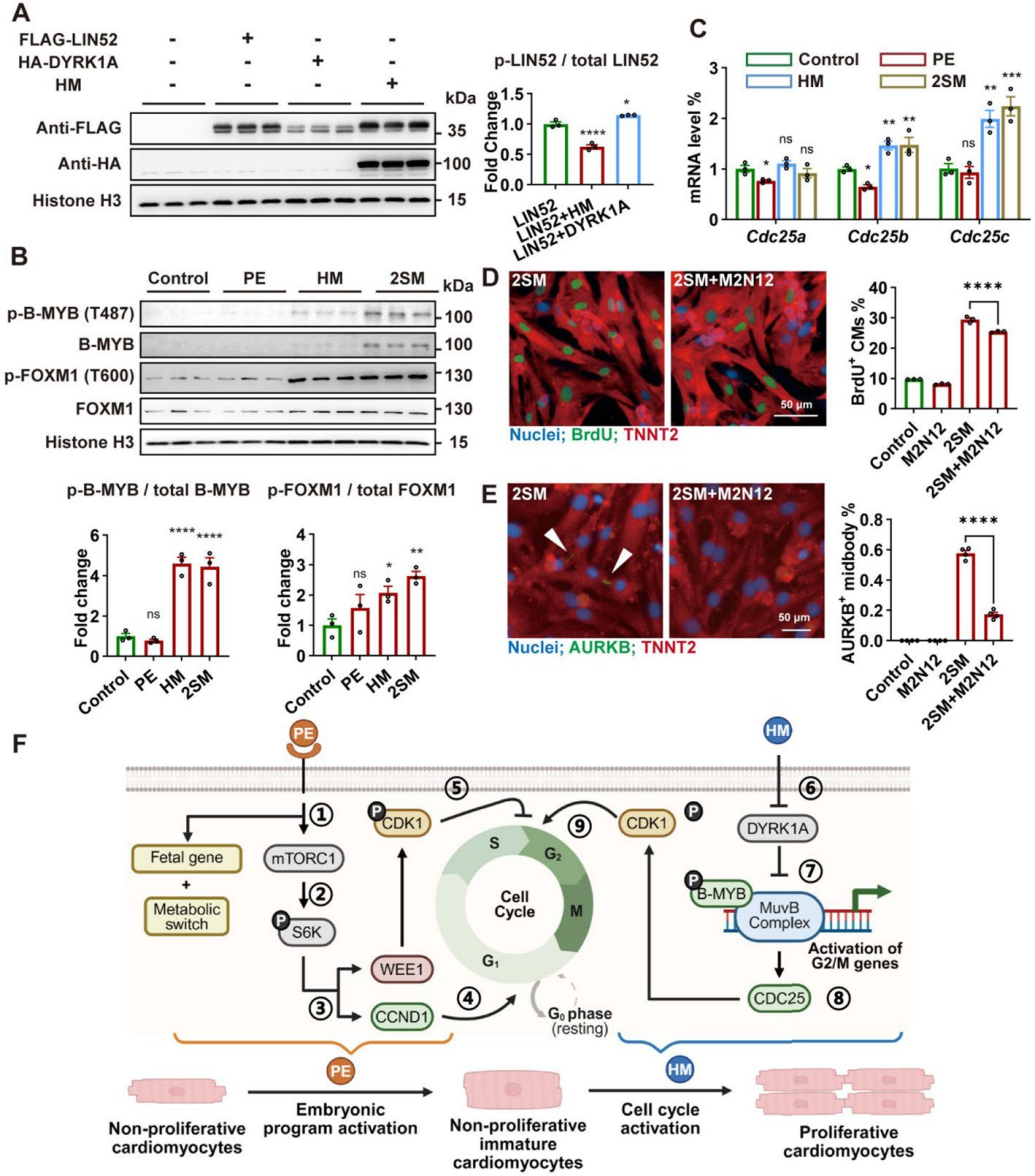
HM promotes G2/M gene expression and cytokinesis through activation of the MMB and FOXM1-MuvB complexes. **A** Western blot analysis of LIN52 phosphorylation in NRVMs transfected with FLAG-LIN52, with HM treatment or HA-DYRK1A overexpression. Bar graph shows quantification of phosphorylated LIN52 relative to total LIN52. The upper band of FLAG-LIN52 protein in the western blots represents the phosphorylated FLAG-LIN52. Data are normalized to Histone H3 and presented as fold change relative to control. *n* = 3 per group. All data represent mean ± SEM, with individual data points shown as dots overlaid on the bar graphs. Statistical significance was determined by one-way ANOVA (*P < 0.05, ****P < 0.0001). **B** Western blot analysis of phosphorylated and total B-MYB and FOXM1 in NRVMs treated with DMSO (Control), PE, HM, or 2SM for 24 h. Bar graphs show quantification of p-B-MYB/B-MYB and p-FOXM1/FOXM1 ratios. Data are normalized to Histone H3 and presented as fold change relative to control. *n* = 3 per group. All data represent mean ± SEM, with individual data points shown as dots overlaid on the bar graphs. Statistical significance was determined by one-way ANOVA (ns, not significant; *P < 0.05, **P < 0.01, ****P < 0.0001). **C** qRT-PCR analysis of *Cdc25a*, *Cdc25b*, and *Cdc25c* mRNA levels in NRVMs treated with DMSO (Control), PE, HM, or 2SM for 24 h. Data are normalized to *Hprt1* and presented as relative expression levels to control. *n* = 3 per group. All data represent mean ± SEM, with individual data points shown as dots overlaid on the bar graphs. Statistical significance was determined by one-way ANOVA (ns, not significant; *P < 0.05, **P < 0.01, ***P < 0.001). **D** and **E** Representative immunofluorescence images and quantification of BrdU-positive (**D**) and AURKB-positive midbody (**E**) cardiomyocytes after 2SM treatment with or without CDC25 inhibitor M2N12 for 48 h. BrdU: *n* = 3 per group. AURKB: *n* = 4 per group. Scale bars, 50 μm. All data represent mean ± SEM, with individual data points shown as dots overlaid on the bar graphs. Statistical significance was determined by one-way ANOVA (****P < 0.0001). **F** Working model illustrating the sequential mechanism by which PE and HM promote cardiomyocyte cell-cycle progression. (1) PE activates a fetal-like and hypertrophy-associated transcriptional priming program. (2) PE activates the mTORC1-S6K signaling pathway. (3) Activation of this pathway contributes to the upregulation of CCND1, thereby promoting G1/S transition and cell-cycle entry. (4) Activation of this pathway also increases WEE1 expression. (5) WEE1 phosphorylates CDK1 and imposes a G2/M checkpoint block, preventing completion of mitotic progression. (6) HM inhibits DYRK1A activity and reduces DYRK1A-mediated LIN52 phosphorylation. (7) Reduced LIN52 phosphorylation facilitates activation of the MMB and FOXM1-MuvB complexes. (8) Activated MMB and FOXM1-MuvB complexes induce G2/M-phase gene expression, including CDC25 phosphatases. (9) CDC25-mediated CDK1 dephosphorylation relieves the PE-induced G2/M arrest, enabling G2/M progression and cytokinesis

To test this hypothesis, we first examined the phosphorylation status of LIN52 in the context of exogenous LIN52 expression. Specifically, we assessed how DYRK1A inhibition or overexpression affects LIN52 phosphorylation. Western blot analysis revealed that overexpression of DYRK1A significantly increased LIN52 phosphorylation. In contrast, treatment with HM reduced LIN52 phosphorylation (Fig. 6A). We then assessed the activation of MMB complex and FOXM1-MuvB complex. HM and 2SM treatment enhanced both phosphorylation and total levels of B-MYB and FOXM1, whereas PE alone had limited impact (Fig. 6B). To avoid potential off-target effects associated with pharmacological inhibition by HM, we next performed genetic validation using cardiomyocyte-specific *Dyrk1a* conditional knockout (*Dyrk1a* cKO) mice. Western blot analysis confirmed efficient reduction of DYRK1A protein levels in *Dyrk1a* cKO mice. Notably, *Dyrk1a* deletion increased the phosphorylation levels of both FOXM1 and B-MYB (Fig. S12), providing genetic evidence that inhibition of DYRK1A activates the B-MYB/FOXM1 axis. These findings indicate that HM inhibits the activity of DYRK1A and leads to the activation of the MMB and FOXM1-MuvB complex.

The activation of MMB complex and FOXM1-MuvB complex enhances the transcription level of G2/M phase genes (Fischer and Müller 2017). qRT-PCR results confirmed that HM and 2SM significantly up-regulated G2/M phase genes transcripts, including *Mybl2*, *Foxm1*, *Cdk1*, *Ccnb1*, *Cdc25b* and *Cdc25c* (Fig. 6C, S13). Given the critical role of the CDC25 phosphatase family in promoting CDK1 dephosphorylation and G2/M progression, we hypothesized that HM-mediated upregulation of CDC25 genes may be essential for relieving PE-induced G2/M arrest. To test this, we co-treated cardiomyocytes with the CDC25 inhibitor M2N12 and 2SM. Western blot analysis showed that M2N12 treatment markedly increased CDK1 phosphorylation levels in 2SM-treated cardiomyocytes (Fig. S14). Furthermore, we found that M2N12 significantly suppressed 2SM-induced increases in BrdU^+^ cardiomyocytes, pH3^+^ cardiomyocytes, and AURKB^+^ midbody formation (Fig. 6D-E; S15). Notably, the inhibitory effect of M2N12 was more pronounced on G2/M markers (pH3 and AURKB) than on BrdU incorporation, suggesting that CDC25 activity is more directly involved in the G2/M transition than in S-phase entry. These results indicate that CDC25 activity is required for CDK1 activation and G2/M progression in the context of 2SM treatment.

Taken together, our findings support a model in which HM promotes cardiomyocyte G2/M phase entry by inhibiting DYRK1A, thereby activating MMB and FOXM1-MuvB complexes and upregulating G2/M genes including the CDC25 genes. CDC25 phosphatases, in turn, dephosphorylate CDK1 and enable successful completion of the G2/M transition, effectively relieving the G2/M arrest by PE (Fig. 6F).

## Discussion

We have previously reported that the five-small-molecule cocktail (5SM) effectively promoted cardiomyocyte proliferation and heart regeneration after rat MI (Du et al. 2022). However, its complexity presents barriers to both the molecular mechanisms of chemical action and their clinical translation. Here, we identified a dual-compound combination, 2SM, comprising PE and HM, from the 5SM cocktail, which retains substantial regenerative capacity of 5SM in promoting cardiomyocyte proliferation and heart regeneration after MI. This simplified cocktail allows us to gain novel insights into the chemical-induced cell-cycle reentry and cell division, as well as provides a novel therapeutic regimen for myocardial infarction.

PE, a widely used α1-adrenergic receptor agonist, has long been associated with cardiomyocyte hypertrophy (Cotecchia et al. 2015). Intriguingly, our current findings highlight an additional role for PE in promoting cardiomyocyte re-entry into the cell cycle. This novel regulation of the cell cycle by PE primarily involves activation of the mTOR signaling pathway, resulting in upregulation of CCND1 and WEE1 kinase expression. Consistent with previous studies, exogenous overexpression of CCND1 alone has been reported to enhance cardiomyocyte DNA synthesis and multinucleation, while upregulation of WEE1 was found to restrict cardiomyocyte proliferation; these observations align closely with the cellular phenotypes we observed following PE treatment (Soonpaa et al. 1997; Tampakakis et al. 2021). Interestingly, mTOR activation alone fails to fully induce cardiomyocyte cell cycle re-entry. We postulate that this limitation may be related to a concurrent embryonic transcriptional program that PE reactivates. In detail, our transcriptional data demonstrate that PE treatment leads to significant upregulation of fetal genes and downregulation of genes involved in fatty acid oxidation, indicative of a shift from postnatal metabolic and transcriptional states towards embryonic-like immature cardiomyocytes. Recent studies indicate that fetal gene activation (e.g., *Nppa*, *Nppb*, *Myh7*), traditionally associated with hypertrophy, may also prime cardiomyocytes for dedifferentiation and proliferation (Chen et al. 2021; Li et al. 2023). Therefore, the PE-induced reactivation of fetal gene programs, together with mTOR pathway activation, collectively primes cardiomyocytes by initiating the immature state and biosynthetic preparations for subsequent cell division.

Several studies have implicated DYRK1A as a negative regulator of cardiomyocyte proliferation during heart regeneration, where genetic deletion of Dyrk1a has been shown to enhance cardiomyocyte proliferation (Lan et al. 2022; Young et al. 2022). In our study, however, we found that while the DYRK1A inhibitor HM significantly upregulated the expression of cell cycle related genes, its ability to promote cytokinesis was relatively limited. We speculate that this difference might be caused by the varying efficiencies between genetic knockout and chemical inhibition in suppressing DYRK1A activity. Additionally, HM itself appears to have a moderate effect on promoting cardiomyocyte proliferation, which could further explain why different studies have reported varied results. Importantly, the combination of PE and HM induced a markedly stronger proliferative response in cardiomyocytes compared to either compound alone. Moreover, small-molecule-based strategies offer a more clinically feasible and reversible alternative to genetic manipulation. More studies are needed to clarify how DYRK1A inhibition influences the efficiency of cardiomyocyte proliferation.

The synergistic pro-proliferative effect of PE and HM relies on a stepwise coordination of distinct phases of the cardiomyocyte cell cycle. While previous studies have demonstrated that overexpression of four cell cycle regulators, including G1/S-phase proteins CCND1 and CDK4, and G2/M-phase proteins CCNB1 and CDK1, can drive cardiomyocyte division (Mohamed et al. 2018), our findings reveal that such sequential control can be achieved through just two small molecules. This regulation process also represents a novel model of cardiomyocyte proliferation, wherein cells first undergo a transition into an embryonic, non-proliferative hypertrophic-like state characterized by activation of fetal programs, a metabolic shift from fatty acid oxidation to glycolysis, and enhanced protein biosynthesis. This intermediate state appears to provide the necessary permissive environment and molecular resources for cell cycle progression. Subsequently, further activation of specific cell cycle proteins enables successful completion of mitosis and cytokinesis. In summary, this study establishes a basis for the clinical translation of 2SM and provides new insights into the molecular mechanisms underlying cardiomyocyte proliferation, highlighting 2SM as a promising therapeutic approach for ischemic heart disease.

Compared to gene therapies or complex multi-drug regimens, the simplified 2SM combination provides precise temporal control, streamlined formulation, and improved regulatory feasibility. Its efficacy in promoting cardiac functional recovery after myocardial infarction, without inducing pathological hypertrophy or systemic toxicity, underscores its favorable therapeutic profile. Furthermore, the minimal nature of this approach makes it particularly well-suited for integration with advanced, tissue-specific delivery technologies, such as photo-crosslinkable hydrogels or cardiac-targeted nanoparticles, to achieve localized and sustained therapeutic effects within injured cardiac tissue.

Despite these promising findings, our study has several limitations. First, given that different cardiomyocyte subpopulations contribute distinct functions during cardiac regeneration (Wang et al. 2020), and that proliferating cardiomyocytes often show region-specific distribution (Liu et al. 2021b), clarifying these features will be important for understanding the precise regenerative effects of 2SM. Single-cell and spatial transcriptomic technologies provide powerful tools to resolve cellular heterogeneity and spatial dynamics (Tang et al. 2025), and thus, future studies using these approaches will help reveal how 2SM influences different cell types and the dynamic responses of proliferative cardiomyocytes during heart regeneration. Second, our results also suggest a possible link between cardiomyocyte hypertrophy and proliferation. Since NRG1/ERBB2 signaling pathway and Hippo signaling pathway are known to play roles in cardiomyocyte dedifferentiation (D’Uva et al. 2015; Ikeda et al. 2019), it will be important to investigate whether these pathways are also involved in 2SM-induced cardiomyocyte proliferation. Third, the mechanistic experiments in the present study were mainly performed using NRVMs. Neonatal CMs retain a higher intrinsic proliferative capacity than adult CMs; the detailed mechanisms of 2SM in adult CMs, human CMs, and the in vivo cardiac environment require further investigation. Finally, further medicinal chemistry studies that target the α-adrenergic receptor and DYRK1A kinase will discover both safe and effective clinical drugs for heart disease.

## Materials and methods

### Experimental animals

Neonatal (postnatal day 3, P3) and adult (8-week-old) wild-type Sprague–Dawley (SD) rats were purchased from Vital River Laboratory Animal Technology Co., Ltd. (Beijing, China). *Dyrk1a*^flox/flox^ mice were kindly provided by Dr. Chunyu Zeng (The Third Military Medical University). MADM-ML-11^TG^ and MADM-ML-11^GT^ mouse lines were kindly provided by Dr. Chong Liu (Zhejiang University School of Medicine). MADM-ML-11^TG/GT^ heterozygous mice were generated by crossing MADM-ML-11^TG^ and MADM-ML-11^GT^ homozygotes. Cardiac-specific MADM mice were obtained by crossing αMHC-MerCreMer; MADM-ML-11^TG^ mice with αMHC-MerCreMer; MADM-ML-11^GT^ mice as described (Du et al. 2022). All animal procedures were approved by the Institutional Animal Care and Use Committee of Peking University and conducted in accordance with institutional and national ethical guidelines.

### Isolation of primary NRVMs

NRVMs were isolated from P3 SD rats as previously described (Du et al. 2022). Briefly, ventricles were subjected to enzymatic digestion in calcium- and magnesium-free Hank’s Balanced Salt Solution (Macgene, CC016) containing 1 mg/mL trypsin (Amresco, VWRV0785) and 0.5 mg/mL collagenase type II (Gibco, 17,101,015) at 37 ℃. The tissues were digested for 8 cycles, 5 min each, and cell suspensions were neutralized with DMEM high-glucose medium supplemented with 10% fetal bovine serum (FBS, Yeasen, 4013ES76). Cells were collected by centrifugation at 200 × *g* for 5 min. The resulting cell pellet was resuspended in DMEM high-glucose medium containing 10% FBS and 1% penicillin–streptomycin, and pre-plated in 10-cm culture dishes for 2 h at 37 ℃ in a 5% CO_2_ incubator to remove non-cardiomyocytes by differential adhesion. Non-adherent cardiomyocytes were then collected by gentle aspiration, centrifuged (200 × g, 5 min), and resuspended in complete culture medium containing 20 μM cytosine β-D-arabinofuranoside (Vetec, V900339) to suppress fibroblast proliferation. Cells were adjusted to a final density of 1 × 10^6^ cells/mL in appropriate culture plates and maintained at 37 ℃ with 5% CO_2_ for 48 h before experimental treatments.

MADM cardiomyocytes were isolated from P3 MADM mice. Briefly, hearts were collected in calcium- and magnesium-free HBSS and digested overnight at 4 °C in 0.25% trypsin–EDTA (Solarbio, T1320). On the following day, tissues were serially digested in 0.5 mg/mL collagenase type II with 5 mg/mL BSA (GPC, AA671) at 37 ℃ for two 15-min cycles. Cell suspensions were pooled, centrifuged at 200 × *g* for 5 min, and resuspended in DMEM high-glucose medium supplemented with 10% FBS and 1% penicillin–streptomycin. Cells were plated for 2 h to allow differential adhesion and then collected non-adherent cardiomyocytes for downstream experiments.

### Adenoviral infection

Adenovirus pAdeno-Tnnt2-mAG-hGeminin(1–110), pAdeno-Tnnt2-Cre, pAdeno-CMV-Rheb, pAdeno-CMV-*rDyrk1a*-HA, and pAdeno-CMV-3xFlag-*rLin52*-V5 were packaged by OBiO Technology (Shanghai, China). For infection, adenovirus stocks were diluted in DMEM containing 1% FBS and 1% penicillin–streptomycin to a final multiplicity of infection (MOI) of 10, and then added to cell cultures. Cells were infected at 2 days after isolation. Phenotypic or molecular assays were performed at 48–96 h post-infection.

### Chemical treatment of cultured cardiomyocytes

Compounds and their working concentrations were as follows: phenylephrine hydrochloride (PE, TargetMol, T0453), 2 μmol/L; harmine (HM, MCE, HYN0737A), 5 μmol/L; baricitinib (BA, TargetMol, T2485), 4 μmol/L; Vo-ohpic trihydrate (VO, MCE, HY13074), 2 μmol/L; AZD3965 (AZ, MCE, HY12750), 2 μmol/L; rapamycin (MCE, HY10219), 20 nmol/L; and M2N12 (MCE, HY128769), 0.5 μmol/L. Compounds were diluted in DMEM containing 0.5% FBS and 1% penicillin–streptomycin, and added to cardiomyocyte cultures. Cells were collected for phenotypic or molecular analyses 24–48 h after treatment. For cell cycle-related experiments, cardiomyocytes were serum-starved in 0.5% FBS DMEM for 24 h prior to compound treatment. The working concentrations of PE, HM, BA, VO, and AZ were selected based on our previous optimization experiments (Du et al. 2022), in which a concentration range of 0.5—10 μmol/L was tested to identify the doses with the strongest pro-proliferative effects on cardiomyocytes. For the other compounds, working concentrations were determined according to the manufacturers’ recommendations and further optimized using concentration-gradient testing.

### hiPSC-CM culture and treatment

Human ventricular cardiomyocytes derived from induced pluripotent stem cells (hiPSC-CMs) were obtained from Cosmos Biotech (Nanjing, China; Cat# CSM-H230011). Culture plates were pre-coated with cardiomyocyte plating medium (Cosmos Biotech, CSM-H230014) and incubated at 37 °C for 1 h to ensure even coverage of the surface. Cryopreserved cells were thawed using the cardiomyocyte recovery medium (CSM-H230012) according to the manufacturer’s instructions and seeded onto the pre-coated 96-well plates at a density of 8 × 10^4^ cells per well. Cells were maintained in cardiomyocyte maintenance medium (CSM-H230015) at 37 °C in a humidified atmosphere of 5% CO₂. The medium was left unchanged for the first 48 h after recovery and replaced every 48 h thereafter. Beating hiPSC-CMs were then treated with 2SM comprising PE (2 μmol/L) and HM (5 μmol/L), or an equal volume of DMSO as the vehicle control for 48 h.

### Immunofluorescence staining of cultured NRVMs

NRVMs were washed three times with ice-cold PBS and fixed in 4% paraformaldehyde (PFA) at room temperature for 12–15 min. After fixation, cells were washed three times with PBS and permeabilized and blocked in PBS containing 1% BSA and 0.1% Triton X-100 for 1 h at room temperature. Primary antibodies diluted in blocking buffer were added and incubated overnight at 4 °C. The following day, cells were returned to room temperature for 30 min and washed three times with PBST (PBS +0.1% Tween-20). Fluorescent secondary antibodies diluted in blocking buffer were added and incubated for 1 h at room temperature in the dark. After washing three times with PBST, nuclei were stained with Hoechst 33,342 (Solarbio, C0031, 1 μg/mL) for 15 min. Cells were then washed and imaged in PBS. The following antibodies were used: rabbit anti-KI67 (Abcam, ab15580; 1:500), mouse anti-cardiac Troponin T (Abcam, ab8295; 1:300), rabbit anti-phospho-Histone H3 (Ser10) (CST, 9701; 1:300), rabbit anti-Aurora B (CST, 3094; 1:300); Alexa Fluor 488 goat anti-rabbit IgG (Thermo Fisher, A32731; 1:500), and Alexa Fluor 555 goat anti-mouse IgG (Thermo Fisher, A21422; 1:500). Imaging was performed using the Operetta CLS high-content imaging system or Opera Phenix high-content imaging system (PerkinElmer, USA), and phenotypic analysis was carried out with Harmony 5.3 software (PerkinElmer).

### BrdU incorporation and immunofluorescence

For BrdU incorporation assays, NRVMs were serum-starved in DMEM containing 0.5% FBS and 1% penicillin–streptomycin for 24 h, and then incubated with 10 µM BrdU (MCE, HY15910) together with test compounds for 48 h. After treatment, cells were fixed in 4% PFA, permeabilized with 0.1% Triton X-100, and subjected to DNA denaturation with 2 N HCl for 20 min at room temperature, followed by neutralization in 1 M Na_2_B_2_O_4_ for 20 min. After washing, cells were blocked in PBS containing 1% BSA and incubated with rat anti-BrdU antibody (Abcam, ab6326; 1:500) overnight at 4 °C. The following day, Alexa Fluor-conjugated secondary antibodies were applied for 1 h at room temperature, nuclei were counterstained with Hoechst 33,342 (1 µg/mL), and images were acquired using the Operetta CLS high-content imaging system (PerkinElmer). Quantification of BrdU-positive cardiomyocytes was performed with Harmony 5.3 software (PerkinElmer).

### Analysis of cardiomyocyte nucleation and ploidy from adult rat hearts

Adult rat ventricular tissues were fixed in 4% paraformaldehyde for at least two days, followed by incubation in 50% (w/v) potassium hydroxide solution overnight. After a brief wash with PBS, the tissues were gently crushed to release dissociated cardiomyocytes. Cells were washed three times with PBS, diluted to an appropriate concentration, and seeded into 96-well plates. Nuclei were stained with 4′,6-diamidino-2-phenylindole (DAPI), and images were acquired on a PerkinElmer Opera Phenix high-content imaging system.

For nucleation analysis, the numbers of mono-, bi-, and poly-nucleated cardiomyocytes were determined manually using the Count Tool (ImageJ). At least 200 cardiomyocytes per sample were analyzed. For ploidy analysis, the ploidy of cardiomyocyte nuclei was determined by normalizing the DAPI intensity of cardiomyocyte nuclei to that of non-cardiomyocyte nuclei in the same imaging field, with non-cardiomyocytes assumed to be diploid (2N). The integrated DAPI fluorescence intensity of cardiomyocyte and non-cardiomyocyte nuclei was measured using ImageJ, and cardiomyocyte nuclear values were normalized to the average value of non-cardiomyocyte nuclei in the same field. Nuclei with normalized integrated pixel densities of 0.5–1.5 were classified as 2 N, those of 1.5–2.5 as 4 N, and those above 2.5 as 4 N +. At least 200 cardiomyocytes per sample were analyzed.

### Assessment of cardiomyocyte size in NRVMs

To evaluate the effect of 2SM on cardiomyocyte size in vitro, NRVMs were isolated from 3-day-old Sprague–Dawley rat hearts. Isolated NRVMs were seeded onto 96-well plates and allowed to attach for 48 h. Cells were then treated with 2SM or an equal volume of DMSO as the vehicle control for 48 h. After treatment, immunofluorescence staining was performed as described above, using mouse anti-cardiac troponin T (cTnT, 1:300, Abcam, ab8295) as the primary antibody and anti-mouse IgG (H + L) F(ab')2 fragment (Alexa Fluor 555 conjugate, 1:500, CST, 4409) as the secondary antibody. Nuclei were counterstained with DAPI. Images were acquired on a PerkinElmer Opera Phenix high-content imaging system. Cardiomyocyte size was assessed based on the cTnT⁺ cell area, and the cell surface area was quantified automatically using the Harmony software.

### Total RNA extraction

Total RNA was extracted using TRIzol reagent (Invitrogen, 15596018CN) according to the manufacturer’s protocol with minor modifications. Cells were washed three times with ice-cold PBS, and 1 mL TRIzol was added per 1 × 10^6^ cells. After 5 min incubation on ice, 200 μL chloroform was added per 1 mL TRIzol, mixed thoroughly, and incubated on ice for 10 min. Samples were centrifuged at 12,000 × g for 15 min at 4 °C, and the aqueous phase was transferred to new RNase-free tubes. An equal volume of isopropanol was added, mixed gently, and incubated on ice for 10 min, followed by centrifugation at 12,000 × g for 10 min at 4 °C. The RNA pellet was washed with 1 mL of 75% ethanol, centrifuged at 12,000 × g for 5 min at 4 °C, air-dried for 5 min at room temperature, and resuspended in 20 μL RNase-free water. RNA concentration was measured using a NanoDrop spectrophotometer (Thermo Scientific, USA).

### Bulk RNA-seq and analysis

Total RNA was extracted from P3 NRVMs treated with DMSO or compounds for 24 h by using TRIzol reagent. RNA library preparation, reverse transcription, and sequencing were performed by Novogene (Beijing, China). Libraries were sequenced on the Illumina NovaSeq 6000 platform using paired-end sequencing. Raw sequencing data were subjected to quality control using FastQC (v0.12.1) and Trim Galore (v0.6.10). HISAT2 (v2.2.1) was used to build the genome index and align reads to the Rnor_6.0 reference genome obtained from Ensembl (Kim et al. 2019). Gene expression levels were quantified using featureCounts (v2.0.6) (Liao et al. 2014). Differential expression analysis was conducted with limma (v3.56.2) and edgeR (3.42.4) (Ritchie et al. 2015; Robinson et al. 2010). Genes with *P* < 0.05 were considered differentially expressed. Gene ontology (GO), Kyoto Encyclopedia of Genes and Genomes enrichment analysis, and Gene Set Enrichment Analysis were performed using the clusterProfiler package (v4.9.1.003) (Wu et al. 2021).

The raw sequence data reported in this paper have been deposited in the Genome Sequence Archive (Zhang et al. 2025) in National Genomics Data Center (CNCB-NGDC Members and Partners 2025), China National Center for Bioinformation/Beijing Institute of Genomics, Chinese Academy of Sciences (accession number: CRA033763), which are publicly accessible at https://ngdc.cncb.ac.cn/gsa.

### Reverse transcription and quantitative real-time PCR (qRT-PCR)

Reverse transcription was performed using the HiScript® III RT SuperMix for qPCR (+ gDNA wiper) kit (Vazyme, R323) according to the manufacturer’s instructions. qRT-PCR was performed using ChamQ SYBR qPCR Master Mix (Vazyme, Q711) on a LightCycler 96 system (Roche, USA). cDNA was diluted 1:20 with ddH_2_O before qRT-PCR. Ct values were obtained using the LightCycler 96 software and analyzed using the 2^−ΔΔCt^ method (Livak and Schmittgen 2001). *Hprt1* was used as the internal reference gene. The sequences of primers are shown in Table S4.

### Cell cycle analysis by flow cytometry

Cardiomyocytes were harvested by 0.25% trypsin–EDTA digestion, centrifuged at 200 × g for 5 min, and washed once with ice-cold PBS. Cells were resuspended in 85 μL PBS, and 200 μL of pre-chilled 100% ethanol (stored at −20 °C) was added dropwise while vortexing to avoid cell clumping. Cells were fixed on ice for 30 min, then centrifuged at 500 × g for 5 min and washed twice with PBS. Cells were incubated in a staining buffer containing 40 μg/mL propidium iodide (PI; Solarbio, C0080), 20 μg/mL RNase A (Solarbio, R1030), and 0.1% Triton X-100 in PBS. After centrifugation and removal of the supernatant, cells were resuspended in 150 μL staining buffer and incubated at 37 °C for 15 min in the dark. Samples were then transferred to ice and protected from light before analysis. Flow cytometry was performed on a FACSVerse Flow Cytometer (BD Biosciences, USA). Single live cells were gated using FSC-A and SSC-A parameters. PI fluorescence was detected in the PE channel, and voltages were adjusted to position the G1 and G2/M peaks at ~200 and ~400 intensity units, respectively. At least 10,000 events were acquired per sample. DNA content histograms were analyzed using ModFit LT 5.0 software (Verity Software House) for cell cycle distribution fitting.

### Protein extraction and western blotting

Cells were washed three times with ice-cold PBS and lysed in RIPA buffer (Solarbio, R0010) supplemented with protease and phosphatase inhibitor cocktail (1:50 dilution; Beyotime, P1045) and 1 mmol/L PMSF (Solarbio, P0100). Approximately 100 μL of lysis buffer was added per 1 × 10^6^ cells. Cells were lysed on ice by ultrasonic disruption and centrifuged at 12,000 × g for 15 min at 4 °C. The supernatant was collected, and protein concentration was determined using a BCA protein assay kit (Solarbio, PC0020) according to the manufacturer’s instructions. Protein samples were mixed with 5 × loading buffer (Beyotime, P0015), denatured at 95 °C for 10 min, and separated by 10% SDS-PAGE, followed by immunoblotting. The following primary antibodies were used: rabbit anti-phospho-4E-BP1 (T37/46) (CST, 2855; 1:1,000), rabbit anti-p70 S6 kinase (CST, 2708; 1:1,000), rabbit anti-WEE1 (CST, 13,084; 1:1,000), rabbit anti-phospho-p70 S6 kinase (T389) (CST, 9234; 1:300), rabbit anti-4E-BP1 (CST, 9452; 1:1,000), rabbit anti-HA-tag (Proteintech, 51,064–2-AP; 1:1,000), rabbit anti-phospho-CDK1 (Y15) (CST, 4539; 1:1,000), rabbit anti-CDK1 (HUABIO, ET1607-51; 1:1,000), rabbit anti-phospho-Rb (S807/811) (CST, 8516; 1:1,000), rabbit anti-histone H3.1 (Abmart, P30266F; 1:5,000), rabbit anti-Cyclin D1 (HUABIO, ET1601-31; 1:1,000), rabbit anti-DYKDDDDK tag (Proteintech, 20,534–1-AP; 1:1,000), rabbit anti-B-MYB (Abcam, ab314862; 1:1,000), rabbit anti-phospho-B-MYB (T487) (Abcam, ab76009; 1:1,000), mouse anti-FOXM1 (Santa Cruz, sc-271746; 1:1,000), rabbit anti-phospho-FOXM1 (Thr600) (CST, 14,655; 1:1,000). HRP-conjugated secondary antibodies included goat anti-rabbit IgG-HRP (Bioeasy, BE0101; 1:5,000) and goat anti-mouse IgG-HRP (Bioeasy, BE0102; 1:5,000).

### Cardiac tissue protein extraction and western blotting

Control (*Dyrk1a*^flox/flox^) and cardiomyocyte-specific conditional knockout (αMHC-MerCreMer; *Dyrk1a*^flox/flox^) mice were used. To induce Cre-mediated recombination, tamoxifen (40 mg/kg), dissolved in corn oil, was administered by intraperitoneal injection every other day for a total of 5 doses. Fourteen days after the first injection, whole hearts were harvested for protein extraction. Heart tissues were homogenized and lysed in RIPA buffer (Solarbio, R0010) supplemented with protease and phosphatase inhibitor cocktail (1:50; Beyotime, P1045) and 1 mmol/L PMSF (Solarbio, P0100). Lysates were cleared by centrifugation at 12,000 × g for 15 min at 4 °C, and protein concentration was determined using a BCA protein assay kit (Solarbio, PC0020) according to the manufacturer's instructions. Protein samples were mixed with 5 × loading buffer (Beyotime, P0015), denatured at 95 °C for 10 min, separated by 8% and 12.5% SDS-PAGE, and transferred for immunoblotting. The following primary antibodies were used: rabbit anti-B-MYB (Abcam, ab314862; 1:1,000), rabbit anti-phospho-B-MYB (T487) (Abcam, ab76009; 1:1,000), mouse anti-FOXM1 (Santa Cruz, sc-271746; 1:1,000), rabbit anti-phospho-FOXM1 (Thr600) (CST, 14,655; 1:1,000), rabbit anti-DYRK1A (CST, 8765S; 1:1,000), and mouse anti-alpha Tubulin (Proteintech, HRP-66031; 1:5,000) as a loading control. HRP-conjugated secondary antibodies were goat anti-rabbit IgG-HRP (Bioeasy, BE0101; 1:5,000) and goat anti-mouse IgG-HRP (Bioeasy, BE0102; 1:5,000).

### Metabolite profiling by mass spectrometry

Cardiomyocytes were treated with DMSO or compounds for 24 h, harvested by 0.25% trypsin–EDTA digestion, and centrifuged at 200 × g for 3 min. The cell pellet was washed three times with ice-cold PBS and resuspended in 300 μL of pre-chilled HPLC-grade methanol. Cells were lysed by ultrasonic disruption and centrifuged at 14,000 × g for 10 min at 4 °C. The supernatant was collected and adjusted to a final concentration of 100 mg/mL. For long-chain fatty acid profiling, 40 μL of lysate was mixed with 40 μL ddH_2_O prior to injection. For glycolytic intermediates, 200 μL of lysate was evaporated to dryness and reconstituted in 50 μL ddH_2_O before analysis. Mass spectrometry was performed by the Metabolomics Core Facility at College of Future Technology, Peking University. Automated peak integration was performed using MultiQuant 2.1 and MassHunter software.

### Metabolite profiling of heart tissue by mass spectrometry

Left-ventricular myocardial tissue was dissected from control and 2SM-treated rats at 7 days post-myocardial infarction, snap-frozen, and weighed. Tissues were homogenized in ddH₂O (20 mg tissue per 500 μL), and metabolites were extracted with pre-chilled HPLC-grade methanol followed by centrifugation to collect the supernatant. Long-chain fatty acids were then quantified by mass spectrometry at the Metabolomics Core Facility, College of Future Technology, Peking University.

### Myocardial infarction models

Adult rats or mice were anesthetized by intraperitoneal injection of tribromoethanol (300 mg/kg; Sigma) and connected to a rodent ventilator (Kent Scientific, USA) after confirming full anesthesia. Chest hair was removed, and the skin was disinfected twice with povidone-iodine. A thoracotomy was performed by incising the skin from below the left forelimb to the xiphoid process, followed by blunt dissection of the muscle layer to expose the ribs. The intercostal muscles were cut longitudinally to open the thoracic cavity, and the pericardium was removed to expose the heart. The left anterior descending (LAD) coronary artery was permanently ligated using 6–0 nylon suture. The chest wall was closed by sequentially suturing the muscle and skin layers with 4–0 nylon suture, ensuring complete closure of the thoracic cavity. The surgical site was disinfected with povidone-iodine, and the animal was removed from the ventilator. Penicillin was administered intraperitoneally (10,000 U for rats, 1,000 U for mice), and animals were placed on a 37 °C warming pad until recovery. Chest closure, disinfection, and postoperative care were performed as for those in the MI group.

### Drug delivery

#### GelMA preparation and in situ delivery

GelMA solution (15% w/v) was prepared according to the manufacturer’s instructions (EFL, GM60). Compounds were diluted directly into the GelMA solution to final concentrations of 20 μmol/L PE and 50 μmol/L. The same volume of DMSO was added to the vehicle group. For 5SM, final concentrations were 20 μmol/L PE, 40 μmol/L BA, 50 μmol/L HM, 20 μmol/L VO, and 20 μmol/L. During myocardial infarction surgery, after permanent ligation of the LAD coronary artery, 100 μl of GelMA solution was applied evenly over the infarct and border zones in adult rats, and 80 μL in adult mice. The gel was then crosslinked in situ by exposure to 405 nm light for 15 s. The thoracic cavity was subsequently closed, and standard postoperative care was performed.

#### Intraperitoneal administration of compounds

Compounds were dissolved in 25% (w/v) sulfobutylether-β-cyclodextrin (SBE-β-CD) in saline. For 2SM treatment, adult mice were administered PE (1 mg/kg) and HM (1 mg/kg) by intraperitoneal injection. Adult rats received PE (2 mg/kg) and HM (2 mg/kg) under the same protocol. For 5SM treatment, animals received intraperitoneal injections of PE (2 mg/kg), BA (2 mg/kg), HM (2 mg/kg), VO (10 μg/kg), and AZ (2 mg/kg). Control animals were injected with an equal volume of vehicle containing DMSO in 25% SBE-β-CD saline. The concentrations of compounds incorporated into GelMA and the doses used for intraperitoneal administration were based on our previous laboratory optimization and published work (Du et al. 2022).

#### Tamoxifen administration in MADM mice

Tamoxifen (MCE, HY-13757A) was dissolved in corn oil (MCE, HY-Y1888) at a concentration of 20 mg/mL by shaking overnight at 37 °C. For Cre recombination, adult αMHC-MerCreMer; MADM mice were administered tamoxifen by intraperitoneal injection at a dose of 100 mg/kg once daily for 5 consecutive days. Mice were subjected to experimental procedures 7 days after the final injection.

### Echocardiography assessment

Echocardiography was performed using a Vevo 3100 high-resolution ultrasound system (VisualSonics, Canada). Mice and rats were anesthetized with 3% isoflurane for induction and maintained under 1% isoflurane throughout the procedure. Chest hair was removed, and animals were placed in a supine position on a heated imaging platform with limb electrodes for ECG monitoring. Ultrasound coupling gel was applied to the chest area. A variable-frequency transducer (30 MHz for mice, 20 MHz for rats) was fixed on a motorized holder to acquire two-dimensional short-axis images at the level of the papillary muscles. Once the heart rate was stabilized, the following cardiac parameters were measured: ejection fraction (EF), fractional shortening (FS), left ventricular end-systolic diameter (LVESD), and left ventricular end-diastolic diameter (LVEDD). Data from three consecutive cardiac cycles were analyzed using Vevo LAB software (VisualSonics) according to standard formulas as previously reported (Du et al. 2022).

### Tissue perfusion, collection, and fixation

To prevent coagulation, heparin sodium (10 mg/kg) was administered intraperitoneally 30 min prior to tissue harvest. Animals were then anesthetized with tribromoethanol, and body weight was recorded. Following anesthesia, the abdominal cavity was opened, and the diaphragm was punctured to expose the heart. A 4% PFA solution was perfused via the left ventricle using a syringe, with the left atrial appendage simultaneously incised to allow outflow. Perfusion volume was 20 mL for mice and 50 mL for rats. The heart, liver, spleen, lungs, and kidneys were dissected and rinsed three times with ice-cold PBS. Excess connective tissue was removed, and organs were fixed in fresh 4% PFA at room temperature with gentle agitation. After 1 h, hearts were removed and sectioned transversely into four equally sized parts using a slicing mold; the upper portion containing the left atrial appendage was discarded. Remaining tissues were fixed for an additional 48 h in fresh 4% PFA under gentle agitation at room temperature. Fixed tissues were then dehydrated, paraffin-embedded, and sectioned. Sections were dried in a 37 °C oven for 12 h.

### Immunostaining of heart tissues

Paraffin sections were placed on a slide warmer (Leica HI1220) and incubated at 60 °C for 30 min. Sections were deparaffinized by sequential immersion in xylene (2 ×), 100% ethanol (2 ×), 95%, 85%, 70%, and 50% ethanol, each for 5 min. After three washes in PBS (5 min each) and one rinse in ddH_2_O, antigen retrieval was performed using EDTA buffer with microwave heating for 15 min, followed by cooling to room temperature. Sections were then washed three times in PBS. Blocking solution consisting of PBS supplemented with 10% FBS, 1% DMSO, and 1% Tween-20 was applied and incubated in the dark for 1 h at room temperature. Primary antibodies were diluted in blocking buffer and applied to Sects. (25 μL per section) for overnight incubation at 4 °C (12–16 h). The following antibodies were used: rabbit anti-Ki67 (Abcam, ab15580; 1:500), mouse anti-cardiac Troponin T (Abcam, ab8295; 1:300), and rabbit anti-phospho-Histone H3 (Ser10) (CST, 9701; 1:300).

The next day, sections were washed three times in PBS. Fluorescent secondary antibodies were diluted in blocking buffer and applied to sections for 2 h at room temperature in the dark. The following secondary antibodies were used: Alexa Fluor 488 goat anti-rabbit IgG (Thermo Fisher, A32731; 1:500), Alexa Fluor 488 goat anti-mouse IgG (Thermo Fisher, A11029; 1:500), and Alexa Fluor 555 goat anti-mouse IgG (Thermo Fisher, A21422; 1:500). Sections were then washed three times in PBS. Slides were mounted with DAPI-containing antifade mounting medium. Imaging was performed using the Axio Scan.Z1 microscope (Zeiss, USA). Quantitative analyses were done by counting multiple fields from 3 sections per heart using ImageJ software.

### Masson’s trichrome staining

Masson’s trichrome staining was performed using a commercial staining kit (Solarbio, G1340) according to the manufacturer’s instructions. Slides were mounted using neutral balsam mounting medium after staining. Imaging was performed using the Axio Scan.Z1 microscope (Zeiss, USA). The LV fibrosis area and total LV area in each image were measured using ImageJ, and the fibrosis area is reported as a percentage of the total LV area.

### WGA staining

WGA staining was performed on heart sections collected at 7 and 56 days post-myocardial infarction. After deparaffinization, tissue sections were incubated with 5 μg/mL WGA Oregon Green 488 conjugate (Invitrogen, W6748) in PBS at room temperature for 20 min. Sections were then washed three times with PBS (5 min each) and mounted with DAPI-containing antifade mounting medium. Imaging was performed using the Axio Scan.Z1 microscope (Zeiss, USA). Cross-section areas of cardiomyocytes were measured using ImageJ.

### H&E staining

Paraffin-embedded sections of the heart, liver, spleen, lungs, and kidneys were deparaffinized in xylene and rehydrated through a graded ethanol series. Sections were stained with hematoxylin, differentiated in acid alcohol, and counterstained with eosin, followed by dehydration through graded ethanol, clearing in xylene, and mounting. Tissue morphology was examined to assess histopathological changes, with sections imaged and analyzed using an Olympus VS200 slide scanner.

### Serum ALT and AST measurement

To evaluate the systemic safety of 2SM, adult rats received vehicle or 2SM every other day from day 1 to day 28, followed by weekly administration from day 28 to day 56. Blood was collected on day 56 and serum levels of alanine aminotransferase (ALT) and aspartate aminotransferase (AST) were measured using the Amplex Red Alanine Transaminase Assay Kit (Beyotime, P2711S) and the Amplex Red Aspartate Transaminase Assay Kit (Beyotime, P2715S) according to the manufacturer’s instructions.

### Statistical analysis

All data are presented as mean ± standard error of the mean (SEM). The value of n is mentioned in the figure/figure legends and always stands for separate biological replicates. Statistical analysis was performed using Graphpad Prism 8.3.0. The significance of differences between two groups was determined using unpaired Student’s *t-*test, with two-tailed P values. Among three or more groups, one-way ANOVA analysis (using the Fisher’s LSD test) was used for comparisons. *P* < 0.05 was considered statistically significant and individual P values are mentioned in the figure/figure legends.

## Supplementary Information

Supplementary Material 1. Supplementary figures 1-14; supplementary tables 1-4.

## Data Availability

The datasets used and analysed during the current study are available from the corresponding author on reasonable request. The raw RNA-sequence data reported in this paper have been deposited in the Genome Sequence Archive in National Genomics Data Center, China National Center for Bioinformation/Beijing Institute of Genomics, Chinese Academy of Sciences, under accession number CRA033763. The data are publicly accessible at https://ngdc.cncb.ac.cn/gsa.

## Abbreviations

AARS2: Alanyl-tRNA synthetase 2, mitochondrial
ALT: Alanine aminotransferase
AST: Aspartate aminotransferase
AURKB: Aurora kinase B
BA: Baricitinib
CCNB1: Cyclin B1
CCND1: Cyclin D1
CDC25: Cell division cycle 25
CDK1: Cyclin dependent kinase 1
CDK4: Cyclin dependent kinase 4
CM: Cardiomyocyte
CPT1: Carnitine palmitoyltransferase 1
DEG: Differentially expressed gene
Dpi: Day post injury
DYRK1: Dual-specificity tyrosine phosphorylation-regulated kinase 1
EF: Ejection fraction
Foxm1: Forkhead box M1
FS: Fractional shortening
FUCCI: Fluorescent ubiquitination-based cell cycle indicator
GelMA: Gelatin methacryloyl (methacrylated gelatin)
GO: Gene ontology
GSEA: Gene set enrichment analysis
hiPSC-CM: Human induced pluripotent stem cell-derived cardiomyocyte
HM: Harmine
IHD: Ischemic heart disease
KEGG: Kyoto encyclopedia of genes and genomes
LAD: Left anterior descending
MADM: Mosaic analysis with double markers
MI: Myocardial infarction
mTORC1: Mechanistic target of rapamycin complex 1
Mybl2: MYB proto-oncogene like 2
Nppa: Natriuretic peptide A
Nppb: Natriuretic peptide B
NRVM: Neonatal rat ventricular cardiomyocyte
PCA: Principal components analysis
PE: Phenylephrine hydrochloride
pH3: Phosphorylated histone H3
PPAR: Peroxisome proliferator-activated receptor
P3: Postnatal day 3
qRT-PCR: Reverse transcription and quantitative real-time PCR
RB: Retinoblastoma protein
RNA-seq: RNA sequencing
S6K: S6 kinase
SBE-β-CD: Sulfobutylether-β-cyclodextrin
VO: VO-OHpic trihydrate
WEE1: WEE1 G2 checkpoint kinase
WGA: Wheat germ agglutinin
2SM: Cocktail of two small molecules
4EBP1: EIF4E-binding protein 1
5SM: Cocktail of five small molecules
