# Supplementary material for: Small-molecule cocktail 2SM promotes heart regeneration via inducing embryonic-like cardiomyocyte state
Source: Cell Regen. 2026 Aug 11;15:27. doi: 10.1186/s13619-026-00301-0 (PMC13457617; doi:10.1186/s13619-026-00301-0)
Supplement: Supplementary file 1 [file 13619_2026_301_MOESM1_ESM.docx]

**Figure S1. 2SM promotes cell-cycle activation in human iPSC-derived cardiomyocytes.**

(A and B) Representative images and quantification of pH3-positive (A), KI67-positive (B) human iPSC-derived CMs after treatment with DMSO or 2SM for 48 h. Scale bar, 100 μm. n = 4 per group. Data represent mean ± SEM, with individual data points shown as dots overlaid on the bar graphs. Statistical analysis by Student's *t*-test (**P* < 0.05, ****P* < 0.001).

**Figure S2.** **Continuous 2SM treatment is required for sustaining cardiomyocyte proliferation.**

(A-C) Representative immunofluorescence images and quantification of KI67-positive (A), pH3-positive (B), and AURKB-positive (C) NRVMs following continuous or withdrawn 2SM treatment. NRVMs were treated with 2SM for 48 h and then either maintained in 2SM (2SM- 2SM) or switched to DMSO (2SM-DMSO) for an additional 48 h. NRVMs were treated with DMSO for 96 hours in control group, with medium replacement at the 48 h. Scale bars, 50 μm in (A) and (B), 25 μm in (C). n = 3 per group. Data represent mean ± SEM, with individual data points shown as dots overlaid on the bar graphs. Statistical analysis by one-way ANOVA (ns, not significant; ****P*< 0.001, *****P* < 0.0001).

**Figure S3. 2SM increases adult cardiomyocyte number and promotes a shift toward a mononucleated state.**

(A-D) Assessment of adult cardiomyocyte number, nucleation, and ploidy after 2SM treatment. Adult rat cardiomyocytes were isolated from hearts treated with vehicle or 2SM for 7 days. (A) Representative bright-field images of isolated adult rat cardiomyocytes with DAPI-stained nuclei. Insets show individual mono-nucleated cardiomyocytes. Scale bar, 100 μm. (B) Quantification of total isolated cardiomyocyte number from vehicle- and 2SM-treated hearts. Vehicle: n= 4 animals. 2SM: n = 4 animals. (C) Quantification of the percentage of mononucleated, binucleated, and multinucleated cardiomyocytes. Vehicle, n= 6 animals; 2SM, n = 5 animals. (D) Quantification of the percentage of cardiomyocytes with 2N, 4N, and ≥4N nuclear content. Vehicle: n = 6 animals; 2SM: n = 5 animals. Data represent mean ± SEM, with individual data points shown as dots overlaid on the bar graphs. Statistical analysis by Student's *t*-test (ns, not significant; **P* < 0.05, ***P* < 0.01, ****P* < 0.001).

**Figure S4. 2SM improves cardiac function after MI with efficacy comparable to 5SM in 7 days treatment.**

(A–B) Cardiac function assessed by echocardiography in vehicle-, 2SM-, and 5SM-treated rats following MI. EF% (A) and FS% (B) were measured at baseline, 3 dpi and 7 dpi. Vehicle, n = 7 animals; 2SM, n = 5 animals; 5SM, n = 6 animals. Data represent mean ± SEM, with individual data points shown as dots overlaid on bar graphs. Statistical analysis was performed using one-way ANOVA (ns, not significant; **P* < 0.05, ***P* < 0.01).

**Figure S5. 2SM treatment does not induce pathological hypertrophy or alter morphology of other organs.**

(A) Representative immunofluorescence images and quantification of NRVM cell size after treatment with DMSO or 2SM for 48 h. Cardiomyocytes were identified by TNNT2 staining. Scale bar, 100 μm; n = 5 per group. Data represent mean ± SEM, with individual data points shown as dots overlaid on the bar graphs. Statistical analysis by Student's *t*-test (ns, not significant).

(B) Representative images and quantification of cardiomyocyte cross-sectional area measured by WGA staining at 7 dpi following vehicle or 2SM treatment. Scale bar, 50 µm. Vehicle, n = 10 animals; 2SM, n = 10 animals. Data represent mean ± SEM, with individual data points shown as dots overlaid on the bar graphs. Statistical analysis by Student's *t*-test (ns, not significant).

(C) Representative gross morphology images of liver, spleen, lungs, and kidneys at 56 dpi following vehicle or 2SM treatment. Scale bar, 2 cm.

(D) Quantification of heart weight to body weight (HW/BW) ratio, liver weight to body weight ratio (LiverW/BW), spleen weight to body weight ratio (SW/BW), lung weight to body weight ratio (LungW/BW) and kidney weight to body weight ratio (KW/BW) at 56 dpi. Vehicle, n = 11 animals; 2SM, n = 12 animals. Data represent mean ± SEM, with individual animals are shown as dots overlaid on the bar graphs. Statistical analysis by Student’s *t*-test (ns, not significant).

**Figure S6. 2SM treatment shows no evident systemic toxicity.**

(A) Representative H&E staining of heart, liver, spleen, lung, and kidney sections from vehicle- and 2SM-treated rats at 56 days post-myocardial infarction (56 dpi). Scale bar, 50 μm.

(B and C) Serum alanine aminotransferase (ALT) (B) and aspartate aminotransferase (AST) (C) levels in vehicle- and 2SM-treated rats at 56 dpi. Vehicle, n = 6 animals; 2SM, n = 6 animals. Data represent mean ± SEM, with individual animal shown as dots overlaid on the bar graphs. Statistical analysis was performed using Student’s *t*-test (ns, not significant).

**Figure S7.** **2SM activates the cell-cycle genes while suppresses the PPAR signaling pathway.**

(A) Heatmap showing expression of cell cycle-related genes that were upregulated in 2SM-treated NRVMs. Red arrows showing notable upregulated cell-cycle genes.

(B) Heatmap showing downregulated genes involved in the PPAR signaling pathway. Blue arrows showing notable downregulated PPAR signaling pathway genes. RNA-seq of NRVMs was performed after 24 h treatment with DMSO or 2SM. Genes with adjusted P < 0.05 and |log_2_FC| > 1 are considered significant. n = 3 per group.

**Figure S8. 2SM treatment induces a mild upward trend in myocardial free fatty acid levels.**

Free fatty acid profiling was performed in myocardial tissues from vehicle- and 2SM-treated rats after 7 days of treatment. Relative abundances of individual fatty acid, including FA 22:0, FA 20:0, FA 20:4, FA 18:0, FA 18:1, FA 18:2, FA 16:0, FA 14:0, and FA 14:1, were quantified as fold change relative to control. Vehicle, n = 6; 2SM, n = 5. Data represent mean ± SEM, with individual data points shown as dots overlaid on the bar graphs. Statistical analysis was performed using Student’s *t*-test (ns, not significant; **P* < 0.05, ***P* < 0.01).

**Figure S9.** **PE and HM induce distinct transcriptional programs in cardiomyocytes.**

(A) Principal components analysis (PCA) of gene expression profiles in NRVMs treated with DMSO (Control) or PE for 24 hours.

(B) Volcano plot showing differentially expressed genes in PE-treated versus control NRVMs.

(C) GO enrichment analysis of upregulated and downregulated genes after PE treatment.

(D) Gene set enrichment analysis (GSEA) showing positive enrichment of hypertrophy-associated gene set in PE-treated NRVMs compared with control.

(E) *Myh7*/*Myh6* ratio calculated from RNA-seq data based on RPKM values in DMSO (control) and PE-treated samples. n = 3 per group. Data represent mean ± SEM, with individual data points shown as dots overlaid on the bar graphs. Statistical analysis was performed using Student’s *t*-test (**P* < 0.05).

(F) PCA of gene expression profiles in NRVMs treated with DMSO (Control) or HM for 24 hours.

(G) Volcano plot showing differentially expressed genes in HM-treated versus control NRVMs.

(H) GO enrichment analysis of upregulated and downregulated genes after HM treatment. Genes with adjusted P < 0.05 and |log_2_FC| > 1 are considered significant. n = 3 per group.

**Figure S10. 2SM activates fetal gene expression and increases *Myh7*/*Myh6* ratio in cardiomyocytes.**

(A) Heatmap showing differential expression of genes related to fetal program, including *Myh6*, *Myh7*, *Ezh2*, *Nppa*, and *Nppb*, following DMSO (control) and 2SM treatment.
(B) *Myh7*/*Myh6* ratio calculated from RNA-seq data based on RPKM values in DMSO- (control) and 2SM-treated samples. n = 3 per group. Data represent mean ± SEM, with individual data points shown as dots overlaid on the bar graphs. Statistical analysis was performed using Student’s *t*-test (**P* < 0.05).
(C) GSEA plot showing that the cardiac hypertrophic response gene set was not significantly enriched in 2SM-treated NRVMs compared with controls.

**Figure S11.** **PE but not Rheb overexpression induces fetal gene expression.**

(A) qRT-PCR analysis of fatty acid oxidation genes (*Cpt1a* and *Cpt1b*) in NRVMs treated with PE or RHEB-overexpressing adenovirus.

(B) qRT-PCR analysis of fetal gene markers (*Nppa* and *Nppb*) in the same samples. n = 3 per group. Data represent mean ± SEM, with individual data points shown as dots overlaid on the bar graphs. Statistical analysis by one-way ANOVA (ns, not significant; ***P* < 0.01, ****P* < 0.001, *****P* < 0.0001).

**Figure S12. Cardiomyocyte-specific *Dyrk1a* deletion activates the B-MYB/FOXM1 axis.**

(A) Western blot analysis of DYRK1A, phosphorylated FOXM1 (p-FOXM1, T600), total FOXM1, phosphorylated B-MYB (p-B-MYB, T487), and total B-MYB in *Dyrk1a*^flox/flox^ (Control) and cardiomyocyte-specific *Dyrk1a* conditional knockout (αMHC-MCM; *Dyrk1a*^flox/flox^) mouse hearts. α-Tubulin was used as the loading control.

(B–D) Quantification of DYRK1A protein levels (B), p-FOXM1/total FOXM1 ratio (C), and p-B-MYB/total B-MYB ratio (D) from western blot analysis. Control: n = 3 animals; *Dyrk1a* cKO: n = 3 animals. Data represent mean ± SEM, with individual animals shown as dots overlaid on the bar graphs. Statistical analysis was performed using Student’s *t*-test (***P* < 0.01, ****P* < 0.001, *****P* < 0.0001).

**Figure S13.** **HM and 2SM, but not PE, induce G2/M-related gene expression.**

(A-D) qRT-PCR analysis of G2/M phase genes including *Mybl2* (A), *Foxm1* (B), *Cdk1* (C), and *Ccnb1* (D) in NRVMs after treatment with DMSO, PE, HM, or 2SM for 24 h. Expression levels were normalized to Hprt1 and presented as fold change relative to control. n = 3 per group. Data represent mean ± SEM, with individual data points shown as dots overlaid on the bar graphs. Statistical analysis by one-way ANOVA (ns, not significant; **P* < 0.05, ***P* < 0.01, ****P* < 0.001, *****P* < 0.0001).

**Figure S14.** **CDC25 inhibition increases CDK1 phosphorylation in 2SM-treated cardiomyocytes.**

(A) Western blot analysis of phosphorylated CDK1 (p-CDK1, Y15), total CDK1, and Histone H3 in NRVMs treated with DMSO (Control), CDC25 inhibitor M2N12, 2SM, or 2SM with M2N12 for 48 h.

(B) Quantification of p-CDK1/CDK1 ratio from (A), normalized to Histone H3 and presented as fold change relative to control. n = 3 per group. Data represent mean ± SEM. Statistical analysis by one-way ANOVA (***P* < 0.01).

**Figure S15. CDC25 inhibition impairs G2/M progression in 2SM-treated cardiomyocytes.**

(A and B) Representative image (A) and quantification (B) of pH3-positive NRVMs treated with DMSO (control), M2N12, and 2SM in the presence or absence of M2N12, a CDC25 phosphatase inhibitor. n = 3 per group. Data represent mean ± SEM, with individual data points shown as dots overlaid on the bar graphs. Statistical analysis by one-way ANOVA (*****P* < 0.0001).

**Table S1. Gene Ontology (GO) enrichment analysis of upregulated differentially expressed genes in PE-treated NRVMs.**

Top 10 significantly enriched GO biological process terms among genes upregulated in NRVMs after PE treatment for 24 h

| ID | Description | Count | p.adjust | geneID |
| --- | --- | --- | --- | --- |
| GO:0003012 | muscle system process | 37 | 1.81E-13 | Pde4b/Myh7/Myom2/Stc1/Ptger3/Nppb/Myl4/Cacna1s/Grip2/Myl1/Nr4a1/Nppa/Fdps/Pdlim5/Acta2/Csrp3/Dsc2/Ptgs2/Nr4a3/Kcne1/Trpc3/Adra1a/Synm/Ccn2/Myh1/Myh4/Bdkrb1/Mybpc2/Atp2a1/Ryr3/Stac/Bdkrb2/Htr2b/Gper1/Edn1/Sorbs2/Lmcd1 |
| GO:0006936 | muscle contraction | 30 | 3.67E-12 | Pde4b/Myh7/Myom2/Stc1/Ptger3/Myl4/Cacna1s/Grip2/Myl1/Nr4a1/Nppa/Acta2/Csrp3/Dsc2/Ptgs2/Kcne1/Adra1a/Synm/Ccn2/Myh1/Myh4/Bdkrb1/Mybpc2/Atp2a1/Ryr3/Stac/Bdkrb2/Htr2b/Gper1/Edn1 |
| GO:0006941 | striated muscle contraction | 18 | 4.95E-07 | Pde4b/Myh7/Stc1/Myl4/Cacna1s/Myl1/Nr4a1/Nppa/Csrp3/Dsc2/Kcne1/Adra1a/Synm/Ccn2/Myh1/Atp2a1/Ryr3/Stac |
| GO:0003300 | cardiac muscle hypertrophy | 13 | 2.13E-05 | Myh7/Nppb/Nppa/Fdps/Pdlim5/Csrp3/Nr4a3/Trpc3/Adra1a/Htr2b/Edn1/Sorbs2/Lmcd1 |
| GO:0014897 | striated muscle hypertrophy | 13 | 2.55E-05 | Myh7/Nppb/Nppa/Fdps/Pdlim5/Csrp3/Nr4a3/Trpc3/Adra1a/Htr2b/Edn1/Sorbs2/Lmcd1 |
| GO:0014896 | muscle hypertrophy | 13 | 2.58E-05 | Myh7/Nppb/Nppa/Fdps/Pdlim5/Csrp3/Nr4a3/Trpc3/Adra1a/Htr2b/Edn1/Sorbs2/Lmcd1 |
| GO:0006816 | calcium ion transport | 25 | 3.44E-05 | Pde4b/Crhr2/Stc1/Ptger3/Ramp3/Cacna1s/P2rx5/Nppa/Serpine1/Ptgs2/Kcne1/Trpc3/Adra1a/Ccn2/Bdkrb1/Cacna1i/Lilrb2/Atp2a1/Atp2b2/Ryr3/Stac/Tgfb2/Htr2b/Gper1/Edn1 |
| GO:0003015 | heart process | 18 | 3.81E-05 | Pde4b/Crhr2/Myh7/Stc1/Ramp3/Myl4/Myl1/Nppa/Csrp3/Dsc2/Kcne1/Avpr1a/Adra1a/Ccn2/Atp2a1/Myl7/Tgfb2/Edn1 |
| GO:0060047 | heart contraction | 17 | 7.03E-05 | Pde4b/Crhr2/Myh7/Stc1/Myl4/Myl1/Nppa/Csrp3/Dsc2/Kcne1/Avpr1a/Adra1a/Ccn2/Atp2a1/Myl7/Tgfb2/Edn1 |
| GO:0090257 | regulation of muscle system process | 18 | 7.03E-05 | Pde4b/Myh7/Stc1/Ptger3/Nr4a1/Nppa/Fdps/Dsc2/Ptgs2/Nr4a3/Trpc3/Adra1a/Ccn2/Bdkrb1/Atp2a1/Gper1/Edn1/Lmcd1 |

**Table S2. Gene Ontology (GO) enrichment analysis of downregulated differentially expressed genes in PE-treated NRVMs.**

Top 10 significantly enriched GO biological process terms among genes downregulated in NRVMs after PE treatment for 24 h.

| ID | Description | Count | p.adjust | geneID |
| --- | --- | --- | --- | --- |
| GO:0042572 | retinol metabolic process | 8 | 0.00019 | Ces1e/Ces1d/Ces1f/Rpe65/Retsat/Lipe/Aldh1a1/Lrat |
| GO:0072329 | monocarboxylic acid catabolic process | 11 | 0.00031 | Acot7/Sord/Ces1d/Plin5/Bdh2/Decr1/Lipe/Cpt1a/Acaa2/Hadh/Ech1 |
| GO:0009062 | fatty acid catabolic process | 10 | 0.00031 | Acot7/Ces1d/Plin5/Bdh2/Decr1/Lipe/Cpt1a/Acaa2/Hadh/Ech1 |
| GO:0006721 | terpenoid metabolic process | 9 | 0.00046 | Ces1e/Ces1d/Ces1f/Rpe65/Hmgcs2/Retsat/Lipe/Aldh1a1/Lrat |
| GO:0001523 | retinoid metabolic process | 8 | 0.00067 | Ces1e/Ces1d/Ces1f/Rpe65/Retsat/Lipe/Aldh1a1/Lrat |
| GO:0034308 | primary alcohol metabolic process | 9 | 0.00067 | Ces1e/Ces1d/Ces1f/Bmp5/Rpe65/Retsat/Lipe/Aldh1a1/Lrat |
| GO:0030258 | lipid modification | 12 | 0.00079 | Plppr4/Pla2g7/Plin5/Bdh2/Decr1/Pdk4/Plppr3/Cpt1a/Acaa2/Plpp3/Hadh/Ech1 |
| GO:0016054 | organic acid catabolic process | 13 | 0.00079 | Acot7/Sord/Ces1d/Adhfe1/Plin5/Bdh2/Decr1/Mat1a/Lipe/Cpt1a/Acaa2/Hadh/Ech1 |
| GO:0046395 | carboxylic acid catabolic process | 13 | 0.00079 | Acot7/Sord/Ces1d/Adhfe1/Plin5/Bdh2/Decr1/Mat1a/Lipe/Cpt1a/Acaa2/Hadh/Ech1 |
| GO:0016101 | diterpenoid metabolic process | 8 | 0.00082 | Ces1e/Ces1d/Ces1f/Rpe65/Retsat/Lipe/Aldh1a1/Lrat |

**Table S3. Gene Ontology (GO) enrichment analysis of upregulated differentially expressed genes in HM-treated NRVMs.**

Top 10 significantly enriched GO biological process terms among genes upregulated in NRVMs after HM treatment for 24 h

| ID | Description | Count | p.adjust | geneID |
| --- | --- | --- | --- | --- |
| GO:0007059 | chromosome segregation | 49 | 1.47E-28 | Prc1/Hjurp/Top2a/Tpx2/Aspm/Kif2c/Cenpf/Spag5/Nusap1/Cdca8/Ube2c/Kif22/Ndc80/Fam83d/Cenpe/Aurkb/Bub1/Bub1b/Ect2/Kif18b/Spdl1/Ska1/Fancd2/Kif11/Birc5/Cdca2/Kif15/Kif14/Ncapg/Nuf2/Spc25/Kif4a/Mastl/Brca1/Rad18/Dscc1/Ttk/Haspin/Pttg1/Cenpq/Cenpw/Esco2/Brip1/Nek2l1/Kif18a/Mis18a/Psrc1/Tex15/Mnd1 |
| GO:0098813 | nuclear chromosome segregation | 41 | 1.65E-24 | Prc1/Top2a/Tpx2/Aspm/Kif2c/Cenpf/Spag5/Nusap1/Cdca8/Ube2c/Kif22/Ndc80/Fam83d/Cenpe/Aurkb/Bub1/Bub1b/Ect2/Kif18b/Spdl1/Fancd2/Kif11/Birc5/Kif15/Kif14/Ncapg/Nuf2/Spc25/Kif4a/Mastl/Dscc1/Ttk/Haspin/Pttg1/Cenpq/Esco2/Brip1/Kif18a/Psrc1/Tex15/Mnd1 |
| GO:0000280 | nuclear division | 42 | 8.58E-20 | Prc1/Top2a/Tpx2/Aspm/Kif2c/Spag5/Nusap1/Cdca8/Ube2c/Kif22/Ndc80/Kif20a/Cenpe/Aurkb/Bub1/Bub1b/Kif18b/Spdl1/Fancd2/Mybl1/Kif11/Brme1/Birc5/Cdca2/Kif15/Kif14/Ncapg/Cyp26b1/Nuf2/Kif4a/Mastl/Dscc1/Ttk/Haspin/Pttg1/Esco2/Brip1/Kif18a/Psrc1/Tex15/Rspo1/Mnd1 |
| GO:0000819 | sister chromatid segregation | 30 | 3.58E-18 | Prc1/Top2a/Tpx2/Kif2c/Spag5/Nusap1/Cdca8/Ube2c/Kif22/Ndc80/Cenpe/Aurkb/Bub1/Bub1b/Kif18b/Spdl1/Kif11/Birc5/Kif15/Kif14/Ncapg/Nuf2/Kif4a/Dscc1/Ttk/Haspin/Pttg1/Esco2/Kif18a/Psrc1 |
| GO:0000070 | mitotic sister chromatid segregation | 28 | 5.30E-18 | Prc1/Tpx2/Kif2c/Spag5/Nusap1/Cdca8/Kif22/Ndc80/Cenpe/Aurkb/Bub1/Bub1b/Kif18b/Spdl1/Kif11/Birc5/Kif15/Kif14/Ncapg/Nuf2/Kif4a/Dscc1/Ttk/Haspin/Pttg1/Esco2/Kif18a/Psrc1 |
| GO:0140014 | mitotic nuclear division | 30 | 1.79E-15 | Prc1/Tpx2/Kif2c/Spag5/Nusap1/Cdca8/Ube2c/Kif22/Ndc80/Cenpe/Aurkb/Bub1/Bub1b/Kif18b/Spdl1/Kif11/Birc5/Cdca2/Kif15/Kif14/Ncapg/Nuf2/Kif4a/Dscc1/Ttk/Haspin/Pttg1/Esco2/Kif18a/Psrc1 |
| GO:1903046 | meiotic cell cycle process | 24 | 9.46E-12 | Top2a/Aspm/Cdk1/Ndc80/Kif20a/Cenpe/Bub1/Bub1b/Fancd2/Mybl1/Brme1/Birc5/Cyp26b1/Nuf2/Mastl/Ttk/Haspin/Pttg1/Brip1/Nppc/Kif18a/Tex15/Rspo1/Mnd1 |
| GO:0051310 | metaphase plate congression | 15 | 9.46E-12 | Kif2c/Cenpf/Spag5/Cdca8/Kif22/Ndc80/Fam83d/Cenpe/Aurkb/Spdl1/Kif14/Nuf2/Cenpq/Kif18a/Psrc1 |
| GO:0051303 | establishment of chromosome localization | 16 | 1.26E-11 | Kif2c/Cenpf/Spag5/Cdca8/Kif22/Ndc80/Fam83d/Cenpe/Aurkb/Spdl1/Birc5/Kif14/Nuf2/Cenpq/Kif18a/Psrc1 |
| GO:0140013 | meiotic nuclear division | 22 | 3.45E-11 | Top2a/Aspm/Ndc80/Kif20a/Cenpe/Bub1/Bub1b/Fancd2/Mybl1/Brme1/Birc5/Cyp26b1/Nuf2/Mastl/Ttk/Haspin/Pttg1/Brip1/Kif18a/Tex15/Rspo1/Mnd1 |

**Table S4. Primer sequences used for quantitative real-time PCR (qRT-PCR).** List of forward (F) and reverse (R) primers used for amplification of target genes in NRVMs. Sequences are shown in the 5′ to 3′ direction.

| Primers | Sequence (5′ -3′) |
| --- | --- |
| Rat-*Cpt1a*-F | TCAAACCCATTCGTCTTCTGG |
| Rat-*Cpt1a*-R | CTGCTTATTTTTCCCTCGTGC |
| Rat-*Cpt1b*-F | GTCGCTTCTTCAAGGTTTGG |
| Rat-*Cpt1b*-R | AAGAAAGCAGCACGTTCGAT |
| Rat-*Nppa*-F | ATGGGCTCCTTCTCCATCAC |
| Rat-*Nppa*-R | TCTTCGGTACCGGAAGCTG |
| Rat-*Nppb*-F | GGGCTGTGACGGGCTGAGGTT |
| Rat-*Nppb*-R | AGTTTGTGCTGGAAGATAAGA |
| Rat-*Mybl2*-F | CCTTCTCTCCTTCCCAGTTTCT |
| Rat-*Mybl2*-R | CCATGGAATACTTCTGATCTGGG |
| Rat-*Foxm1*-F | TCAAACAGCGGAACAACTGAA |
| Rat-*Foxm1*-R | ACAAGCTTTGACCCTCACAGG |
| Rat-*Cdk1*-F | GGAACAGAGAGGGTCCGTT |
| Rat-*Cdk1*-R | ATTTCCCGGATTGCCGTACT |
| Rat-*Ccnb1*-F | GGTCGATGTGGAGCAGCATA |
| Rat-*Ccnb1*-R | TGCAGAGTTGGTGTCCATTCA |
| Rat-*Cdc25a*-F | AGGGCTGGGCAGTGACTAT |
| Rat-*Cdc25a*-R | GGCTACACCCCAAGAGCTTC |
| Rat-*Cdc25b*-F | TCTCTGCATGGACTCCCCTA |
| Rat-*Cdc25b*-R | AGCAGCCTCACTGGCAAAG |
| Rat-*Cdc25c*-F | CTATAGCTCCACCTCGCTCG |
| Rat-*Cdc25c*-R | TTTTGGGGTTCCTCCGGACA |
| Rat-*Hprt1*-F | CTCATGGACTGATTATGGACAGGAC |
| Rat-*Hprt1*-R | GCAGGTCAGCAAAGAACTTATAGCC |
